# brainlife.io: a decentralized and open-source cloud platform to support neuroscience research

**DOI:** 10.1038/s41592-024-02237-2

**Published:** 2024-04-11

**Authors:** Soichi Hayashi, Bradley A. Caron, Anibal Sólon Heinsfeld, Sophia Vinci-Booher, Brent McPherson, Daniel N. Bullock, Giulia Bertò, Guiomar Niso, Sandra Hanekamp, Daniel Levitas, Kimberly Ray, Anne MacKenzie, Paolo Avesani, Lindsey Kitchell, Josiah K. Leong, Filipi Nascimento-Silva, Serge Koudoro, Hanna Willis, Jasleen K. Jolly, Derek Pisner, Taylor R. Zuidema, Jan W. Kurzawski, Kyriaki Mikellidou, Aurore Bussalb, Maximilien Chaumon, Nathalie George, Christopher Rorden, Conner Victory, Dheeraj Bhatia, Dogu Baran Aydogan, Fang-Cheng F. Yeh, Franco Delogu, Javier Guaje, Jelle Veraart, Jeremy Fischer, Joshua Faskowitz, Ricardo Fabrega, David Hunt, Shawn McKee, Shawn T. Brown, Stephanie Heyman, Vittorio Iacovella, Amanda F. Mejia, Daniele Marinazzo, R. Cameron Craddock, Emanuale Olivetti, Jamie L. Hanson, Eleftherios Garyfallidis, Dan Stanzione, James Carson, Robert Henschel, David Y. Hancock, Craig A. Stewart, David Schnyer, Damian O. Eke, Russell A. Poldrack, Steffen Bollmann, Ashley Stewart, Holly Bridge, Ilaria Sani, Winrich A. Freiwald, Aina Puce, Nicholas L. Port, Franco Pestilli

**Affiliations:** 1 Indiana University, Bloomington, IN USA; 2 The University of Texas, Austin, TX USA; 3 Vanderbilt University, Nashville, TN USA; 4 McGill University, Montréal, Quebec Canada; 5 Cajal Institute, CSIC, Madrid, Spain; 6 Fondazione Bruno Kessler, Trento, Italy; 7 Applied Physics Laboratory, Johns Hopkins University, Laurel, MD USA; 8 University of Arkansas, Fayetteville, AR USA; 9 University of Oxford, Headington, Oxford, UK; 10 Anglia Ruskin University, Cambridge, UK; 11 New York University, New York, NY USA; 12 University of Limassol, Nicosia, Cyprus; 13 University of Cyprus, Nicosia, Cyprus; 14 Institut du Cerveau, CNRS, Sorbonne Université, Paris, France; 15 University of South Carolina, Columbia, SC USA; 16 Lawrence Technological University, Southfield, MI USA; 17 University of Eastern Finland, Kuopio, Finland; 18 Aalto University School of Science, Espoo, Finland; 19 University of Pittsburgh, Pittsburgh, PA USA; 20 University of Michigan, Ann Arbor, MI USA; 21 Hewlett-Packard Enterprise, Pittsburgh, PA USA; 22 SHEGEL, Massul, Luxembourg; 23 University of Trento, Rovereto, Italy; 24 University of Ghent, Ghent, Belgium; 25 University of Nottingham, Nottingham, UK; 26 Stanford University, Stanford, CA USA; 27 University of Queensland, St Lucia, Queensland Australia; 28 The Rockefeller University, New York, NY USA; 29 University of Geneva, Geneva, Switzerland

**Keywords:** Cognitive neuroscience, Computational neuroscience, Translational research, Technology, Databases

## Abstract

Neuroscience is advancing standardization and tool development to support rigor and transparency. Consequently, data pipeline complexity has increased, hindering FAIR (findable, accessible, interoperable and reusable) access. brainlife.io was developed to democratize neuroimaging research. The platform provides data standardization, management, visualization and processing and automatically tracks the provenance history of thousands of data objects. Here, brainlife.io is described and evaluated for validity, reliability, reproducibility, replicability and scientific utility using four data modalities and 3,200 participants.

## Abstract (web summary)

brainlife.io is a one-stop cloud platform for data management, visualization and analysis in human neuroscience. It is web-based and provides access to a variety of tools in a reproducible and reliable manner.

## Main

Over the past 30 years, neuroimaging has matured to adopt the FAIR (findable, accessible, interoperable and reusable) principles^1,2^, develop reporting best practices^3^ and data standards^4^. While making research more rigorous and transparent, this maturation has inevitably increased compliance requirements. Indeed, just a few years ago it was possible to publish studies with a few hours of data collected and analyzed in a single laboratory. Today, studies require combining hundreds of hours of measurement, across multiple participants, laboratories and data modalities (for example, magnetic resonance imaging (MRI), positron emission tomography, functional near-infrared spectroscopy, electro-encephalography (EEG) and magnetoencephalography (MEG)). To support the needs of a mature neuroimaging field, several data collection efforts have been started; relevant examples are the Human Connectome Project (HCP)^5^, Cambridge Centre for Ageing and Neuroscience study (Cam-CAN)^6^, Adolescent Brain Cognitive Development (ABCD) study^7^, the UK-Biobank^8^, Healthy Brain Network (HBN)^9^, Pediatric Imaging Neurocognition and Genetics (PING) study^10^ and the Natural Scene Dataset^11^. At the same time, the complexity of the data pipeline has also increased with multiple, distinct, software libraries and analysis toolboxes developed^12,13^.

**Fig. 1:**
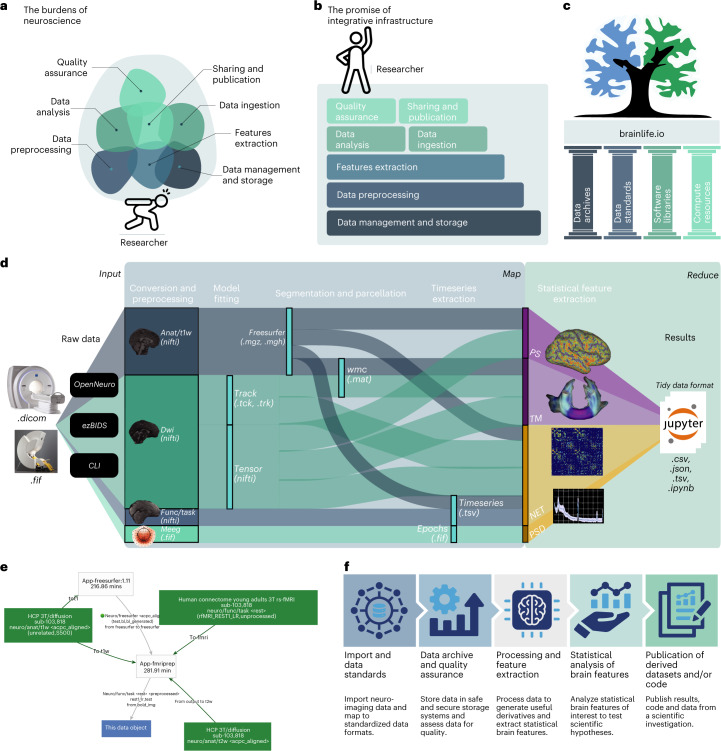
The burdens of neuroscience and the promise of integrative infrastructure. **a**, A figurative representation of the current major burdens of performing neuroimaging investigations. **b**, Our proposal for integrative infrastructure that coordinates services required to perform FAIR, reproducible, rigorous and transparent neuroimaging research thereby lifting the burden from the researcher. **c**, brainlife.io rests on the foundational pillars of the open science community such as data archives, standards, software libraries and compute resources. **d**, brainlife.io’s Map step takes MRI, MEG and EEG data and processes them to extract statistical features of interest. brainlife.io’s reduce step takes the extracted features and serves them to Jupyter Notebooks for statistical analysis. PS, parc-stats datatype; TM, tractmeasures datatype; NET, network datatype and CLI, common line interface. **e**, The brainlife.io technology automates capture of data provenance. All data objects on brainlife.io are stored with a record of the apps, app versions and parameters used to process the data. **f**, The primary services are provided to the user by brainlife.io. Panels **a** and **b** adapted from ref. ^22^ under a Creative Commons license CC BY 4.0.

As compliance requirements grow, so do barriers to entry (Fig. 1a). The mature, neuroimaging field requires increased resources and technical training to piece together and track multiple processes such as data ingestion, standardization, storage, management, preprocessing and feature extraction (Fig. 1a). Currently, no single and low-barrier technology exists to integrate and manage the ever-changing software and data components of a full study. The growing compliance requirements affect the research community inequitably; smaller institutions and lower-income countries are more likely to lack resources and training. As such, this maturation process may risk favoring higher-resourced teams: an outcome that would not only decrease diversity and inclusion, but also slow-down scientific progress.

**Extended Data Fig. 1:**
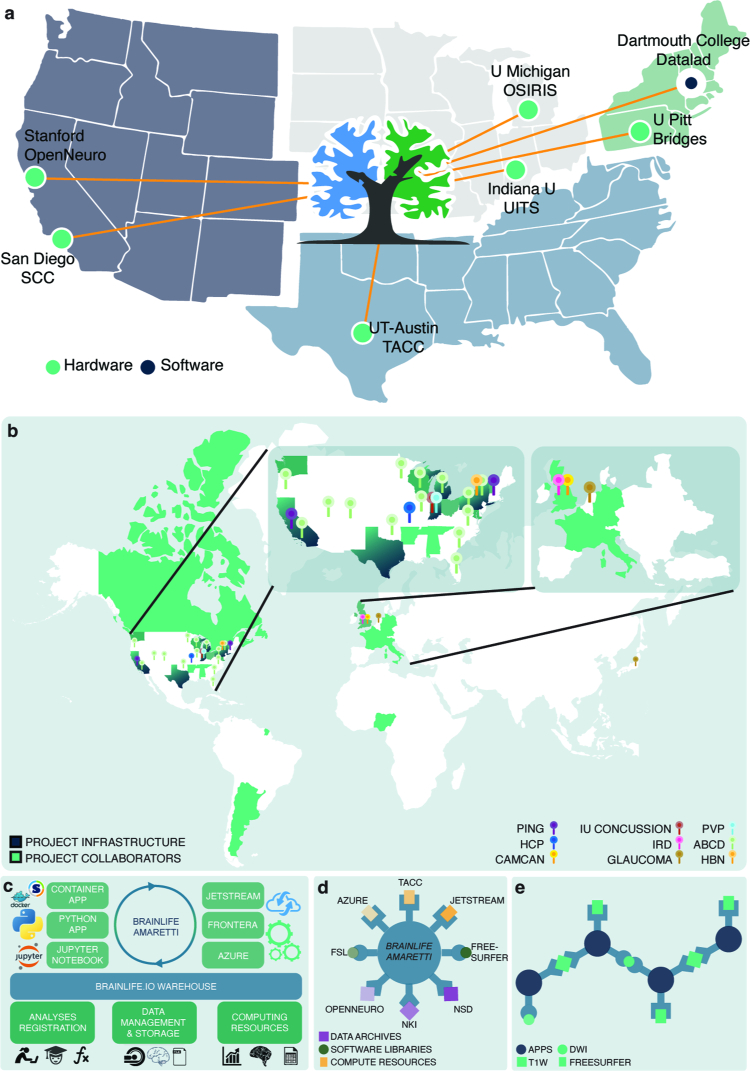
Platform Architecture. **a**. Map of the locations of critical hubs for brainlife.io. **b**. Map the locations of critical facets of this research, including project infrastructure (that is compute resources), collaborators, and data sources. As the United States and Europe are home to many of the infrastructural resources, collaborators, and data sources, more details for these regions are provided (insets). **c**. brainlife.io’s Amaretti links data archives, software libraries, and computing resources. Specifically, ‘Apps’ (containerized services defined on GitHub.com) are automatically matched with data stored in the ‘Warehouse’ with computing resources. Statistical analyses can be implemented using Jupyter Notebooks. **d**. brainlife.io provides efficient docking between data archives, processing apps, and compute resources via a centralized service. **e**. Apps use standardized Datatypes and allow ‘smart docking’ only with compatible data objects. App outputs can be docked by other Apps for further processing.

In support of simplicity, efficiency, transparency and equity in big data neuroscience research, our team has developed a community resource, brainlife.io (Fig. 1b). The brainlife.io platform stands on the pillars of open science (Fig. 1c), to provide free, secure and reproducible neuroscientific data analysis. Because of its web-based availability, brainlife.io should expand opportunities for researchers from nations and institutions with limited research budgets and resources. brainlife.io should then serve as an enabler for researchers and students from all sorts of institutions of higher education and all sorts of backgrounds to access cutting-edge neuroscience analytic tools.

brainlife.io is a ready-to-use and ready-to-expand platform. As a ready-to-use system, it allows researchers to upload and analyze data from MRI, MEG and EEG systems. Data are managed using a secure warehousing system with a proper access-control model. Data can be preprocessed and visualized using version-controlled applications (hereafter referred to as apps; https://brainlife.io/apps; Supplementary Fig. 1), compliant with major data standards (for example, the Brain Imaging Data Structure^4^). As a ready-to-expand system, software developers may submit apps guided by standardization and documentation (https://github.com/brainlife/abcd-spec and https://brainlife.io/docs). The platform uses opportunistic computing to serve commercial and academic clouds to researchers. Computing resources can be registered on brainlife.io for individual users and projects, or the larger community (Extended Data Fig. 1a,b). Supplementary Results 1 describe the technology.

The architecture of brainlife.io is based on a microservice approach for automated and decentralized data management and processing. Microservices are handled by the orchestration system Amaretti (Extended Data Fig. 1c,d and Extended Data Table 1) which deploys computational jobs on high-performance clusters and clouds (for example, Google Cloud, AWS or Microsoft Azure). Data management on brainlife.io is centered around projects, the ‘one-stop-shop’ for data management, processing, analysis and visualization (Supplementary Results 2 and Supplementary Fig. 2). Data archives can be docked by brainlife.io (Extended Data Fig. 1d) and data imported via the portal https://brainlife.io/datasets (Supplementary Table 1). Data from measurement instruments are imported using https://brainlife.io/ezbid (Extended Data Table 1)^14^. Data processing on brainlife.io uses an object-oriented and micro-workflow service model. Data objects are stored using predefined standardized formats, datatypes, that allow automated app pipelining (Extended Data Fig. 1e; https://brainlife.io/datatypes) and provenance tracking for millions of data objects. Data processing Apps are containerized, composable processing units, can be written in any language using containerization technology and are smart, meaning that they automatically identify, accept or reject data objects before processing (Supplementary Results 3 and Supplementary Fig. 3). brainlife.io apps and datatypes are Brain Imaging Data Structure^4^ compatible.

**Extended Data Fig. 2:**
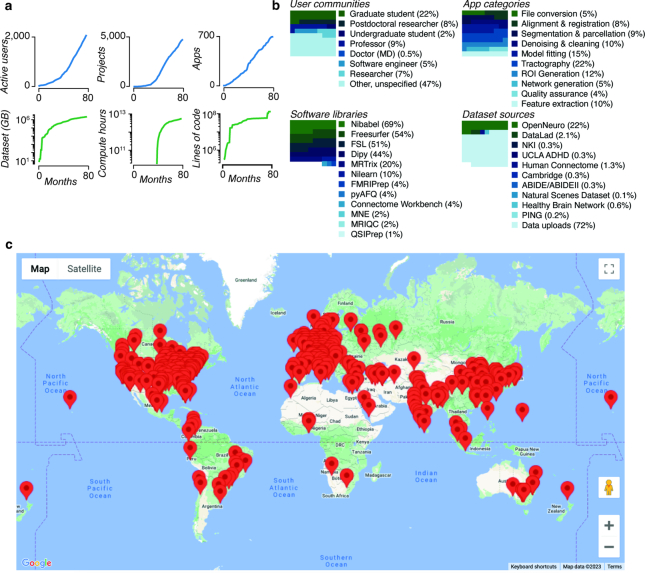
Platform Usage. **a**. Top left. Number of users submitting more than 10 jobs per month. Top middle. Number of projects over time. Top right. Number of Apps over time. Bottom left. Data storage across all Projects. Bottom middle. Compute hours across all Projects (data only available 6 months post project start). Bottom right. Lines of code in the top 50 most-used Apps. **b**. Top left. User communities. Top right. App categories. Bottom left. Percent of total jobs launched with the software library installed (percentage for jobs of top 50 most-used Apps). Bottom right. Datasets sources. **c**. Map of the locations of the users that created an account and accessed brainlife.io. This map is a proxy to the level of attention the platform achieved worldwide.

Complex neuroimaging processing pipelines are simplified into two main steps, akin to Google’s MapReduce algorithm. An initial Map step preprocesses data objects asynchronously, in parallel, to extract features of interest (that is, functional activations, white matter maps, brain networks or time series data; Fig. 1d). A ‘reduce’ step follows where features extracted using apps are made available to preconfigured Jupyter Notebooks to perform analysis and generate figures. Indeed, all analyses and figures in this paper are available in brainlife.io notebooks (Supplementary Table 2). brainlife.io’s data workflow makes it possible to integrate large volumes of data into small sets of features saved into ‘tidy data’ structures (Fig. 1d). For more documentation regarding usage of the platform, see Extended Data Fig. 2 and Supplementary Table 1. Datatypes inform apps allowing automated processing and provenance tracking for millions of data objects. brainlife.io tracks data object IDs, app versions and parameter sets across data processing steps. brainlife.io data provenance graphs visualize (Fig. 1e and Supplementary Table 1) and reproduce (Supplementary Table 1) the data generation steps. brainlife.io lowers the barriers of entry to FAIR neuroimaging by supporting an end-to-end data analysis workflow within a unified ecosystem (Fig. 1f).

**Fig. 2:**
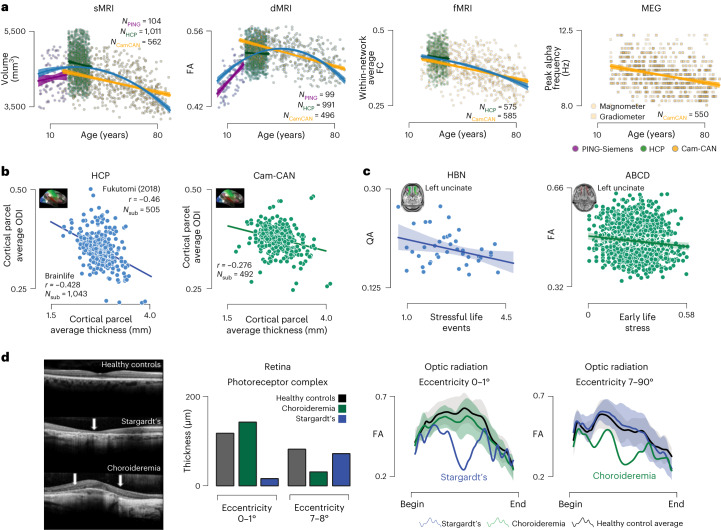
brainlife.io supports scientific discovery and replication. **a–d**, Identifying unique relationships with brain features over the lifespan. **a**, Relationship between participant age and right hippocampal volume, right inferior longitudinal fasciculus (FA, within-network average functional connectivity (FC) derived using the Yeo17 atlas and peak frequency in the alpha band derived from magnetometer (squares) and gradiometers (circles) from MEG data. These analyses include participants from the PING (purple), HCP_1200_ (green) and Cam-CAN (yellow) datasets. Linear regressions were fitted to each dataset, and a quadratic regression was fitted to the entire dataset (blue). **b**,**c**, Replication and generalization of previously reported scientific findings. **b**, Average cortical hcp-mmp parcel thickness (*N*_struc_ = 322) compared to parcel the ODI from the NODDI model mapped to the cortical surface (inset) of the HCPS1200 dataset (*N*_sub_ = 1,043) and Cam-CAN (*N*_sub_ = 492) dataset compared to the parcel-average cortical thickness. **c**, Stressful life events were obtained from the NLES survey from HBN participants (*N*_sub_ = 42) compared to uncinate-average normalized quantitative anisotropy (QA). Mean linear regression (blue line) fits and standard deviation (shaded blue). Early life stress was obtained from multiple surveys collected from ABCD participants (*N*_sub_ = 1,107) compared to uncinate-average FA. Linear regression (green line) fits the data with standard deviation (shaded green). **d**, Identification of clinical biomarkers. **d**, Retinal optical coherence tomography images from healthy controls (top row), patients with Stargardt’s disease (middle row) and patients with Choroideremia (bottom row). From these images, photoreceptor complex thickness was measured for each group (controls, gray; Choroideremia, green; Stargardt’s, blue) in two distinct areas of the retina: the fovea (eccentricities 0–1°) and periphery (eccentricities 7–8°). In addition, optic radiations carrying information for each retinal area were segmented and FA profiles were mapped. Average profiles with standard error (shaded regions) were computed. One participant with Stargardt and one with Choroideremia were identified each having FA profiles that deviated from healthy controls.

**Extended Data Fig. 3:**
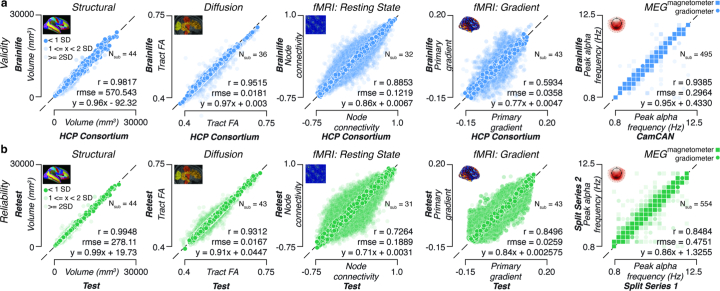
Data processing validity and reliability analysis. Top row (a): Validity measures derived using the HCP Test-Retest (HCP_*TR*_) data. Each dot corresponds to the ratio for a given subject between data preprocessed and provided by the HCP Consortium vs data preprocessed on brainlife.io in a given measure for a given structure. Pearson’s correlation (r), root mean squared error (rmse), and a linear fit between the test and retest results were calculated. Parcel volume (mm^3^). Tract-average fractional anisotropy (FA). Node-wise functional connectivity (FC)*. Primary gradient value derived from resting-state fMRI*. Peak frequency (Hz) in the alpha band derived from MEG. Data from magnetometer sensors are represented as squares, and data from gradiometer sensors are represented as circles. Dark colors represent data within ±1 standard deviation (SD. 50% opacity represents data within 1-2 SD. 25% opacity represents data outside 2 SD. *A representative 5% of data presented. Bottom row (b): Test-retest reliability measures derived from derivatives of the HCP_*TR*_ dataset generated using brainlife.io. Each dot corresponds to the ratio between a test-retest subject and a given measure for a given structure. Pearson’s correlation (r), root mean squared error (rmse), and a linear fit between the test and retest results were calculated.Parcel volume (mm^3^). Tract-average fractional anisotropy (FA). Node-wise functional connectivity (FC)*. Primary gradient value derived from resting-state fMRI*. Peak frequency (Hz) in the alpha band derived from MEG using the Cambridge (Cam-CAN) dataset. Data from magnetometer sensors are represented as squares, and data from gradiometer sensors are represented as circles. Dark colors represent data within ±1 standard deviation (SD. 50% opacity represents data within 1-2 SD. 25% opacity represents data outside 2 SD. *A representative 5% of data presented.

**Extended Data Fig. 4:**
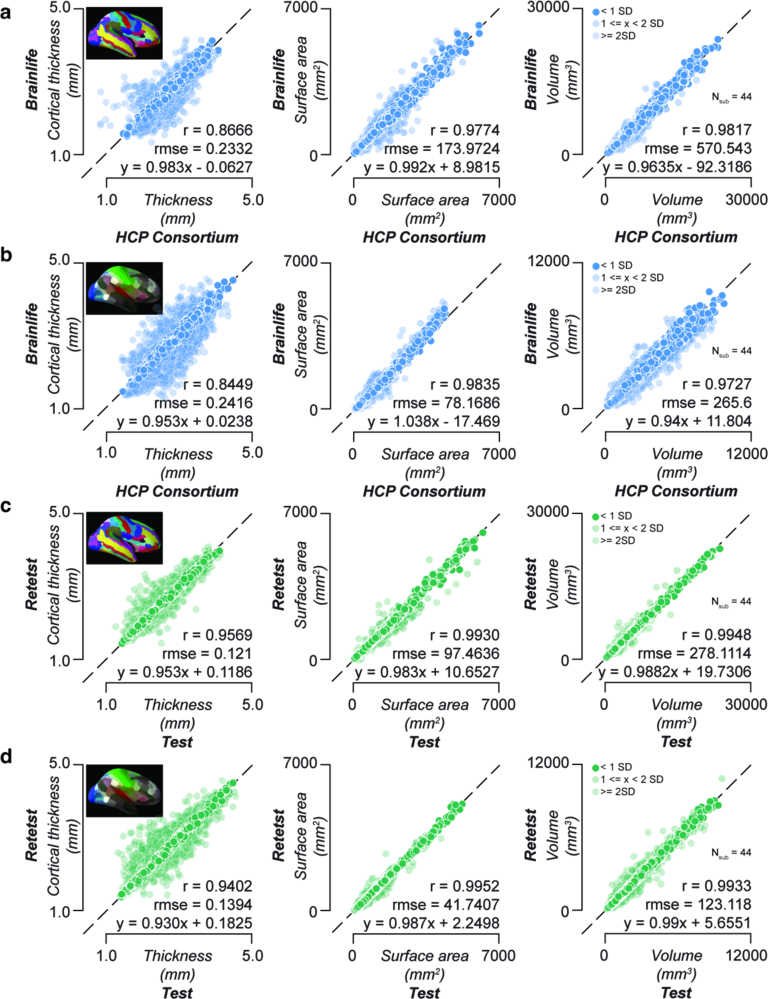
Processing with brainlife.io is valid and test-retest reliability is high - Structural MRI. Top rows: Validity measures derived using the HCP_*TR*_ data preprocessed and provided by the HCP Consortium compared to data preprocessed on brainlife.io. Each dot corresponds to the ratio for a given subject between data preprocessed and provided by the HCP Consortium vs data preprocessed on brainlife.io in a given measure for a given structure. Pearson’s correlation (r), root mean squared error (rmse), and a linear fit between the test and retest results were calculated and provided. **a**. Destrieux Parcel thickness (mm), surface area (mm^2^), and volume (mm^3^). **b**. HCP-mmp Parcel thickness (mm), surface area (mm^2^), and volume (mm^3^). Dark colors represent data within ± 1 standard deviation. 50% opacity represents data within 1-2 standard deviations. 25% opacity represents data outside 2 standard deviations. Bottom rows: Test-retest reliability measures derived from derivatives of the HCPTR dataset generated using brainlife.io. Each dot corresponds to the ratio between a test-retest subject and a given measure for a given structure. Pearson’s correlation (r), root mean squared error (rmse), and a linear fit between the test and retest results were calculated and provided. **c**. Destrieux Parcel thickness (mm), surface area (mm^2^), and volume (mm^3^). **d**. HCP-mmp Parcel thickness (mm), surface area (mm^2^), and volume (mm^3^). Dark colors represent data within ± 1 standard deviation. 50% opacity represents data within 1-2 standard deviations. 25% opacity represents data outside 2 standard deviations.

**Extended Data Fig. 5:**
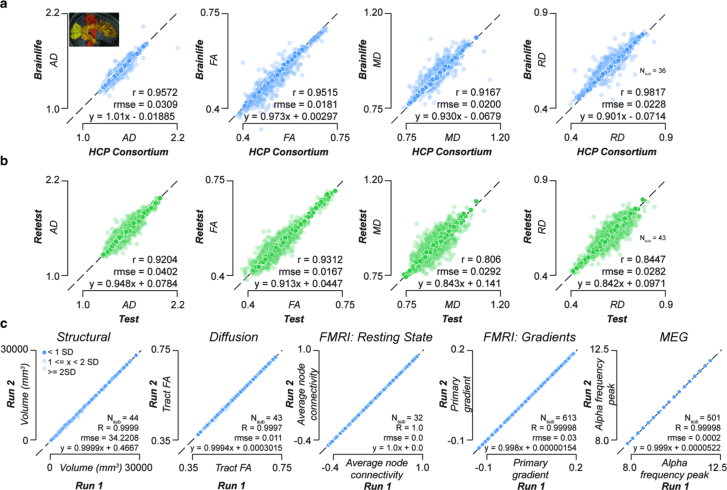
Processing with brainlife.io is valid, reliable, and reproducible. Top row: Validity measures derived using the HCP_*TR*_ data preprocessed and provided by the HCP Consortium compared to data preprocessed on brainlife.io. Each dot corresponds to the ratio for a given subject between data preprocessed and provided by the HCP Consortium vs data preprocessed on brainlife.io in a given measure for a given structure. Pearson’s correlation (r), root mean squared error (rmse), and a linear fit between the test and retest results were calculated and provided. v. Tract average AD, FA, MD, and RD. Dark colors represent data within ±1 standard deviation. 50% opacity represents data within 1-2 standard deviations. 25% opacity represents data outside 2 standard deviations. Bottom row: Test-retest reliability measures derived from derivatives of the HCP_*TR*_ dataset generated using brainlife.io. Each dot corresponds to the ratio between a test-retest subject and a given measure for a given structure. Pearson’s correlation (r), root mean squared error (rmse), and a linear fit between the test and retest results were calculated and provided. w. Tract average AD, FA, MD, and RD. Dark colors represent data within ±1 standard deviation. 50% opacity represents data within 1-2 standard deviations. 25% opacity represents data outside 2 standard deviations. c. Computational reproducibility values derived by repeating runs of brainlife.io Apps using the HCP_*TR*_ dataset and the CAN dataset. Each dot corresponds to the ratio for a given subject between repeated runs of each App for a given structure. Pearson’s correlation (r), root mean squared error (rmse), and a linear fit between the repeated runs was calculated. Destrieux Atlas Parcels volume (mm^3^). Tract-average fractional anisotropy (FA). Node-average functional connectivity (FC). Primary gradient values derived from resting state fMRI. Peak alpha frequency (Hz) in the alpha band derived from MEG.

**Extended Data Fig. 6:**
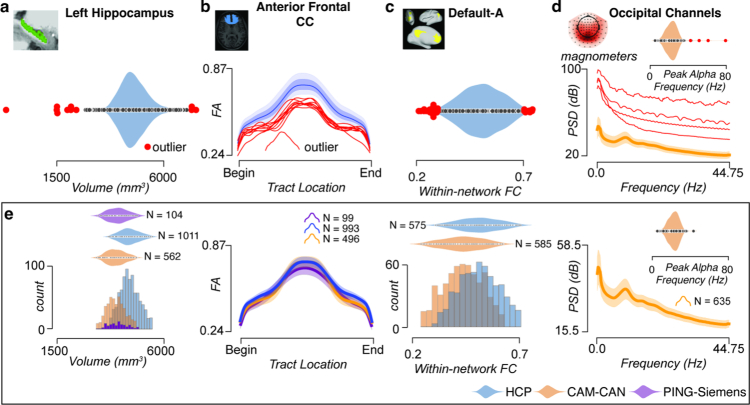
Reference datasets for quality assurance. Example workflow for building normative reference ranges for multiple derived statistical products (cortical parcel volume, white matter tract profilometry, within-network functional connectivity, and power-spectrum density (PSD)). **a**. Cortical volumes of the left hippocampus from HCP participants. Red dots indicate outlier data points. **b**. Average fractional anisotropy (FA) profiles (blue line) plotted with two standard deviations (shaded regions). Red lines indicate outlier profiles. **c**. Within-network functional connectivity for the nodes within the Default-A network using the Yeo17 atlas. Red dots indicate outlier data points. **d**. Average PSD from occipital channels using magnetometer sensors from Cam-CAN participants with one standard deviation (shaded regions). Red lines indicate outlier participants. Peak alpha frequency distribution was also computed, and outliers were detected (inset). **e**. Normative reference distributions for each derived statistical product across the PING (purple), HCP (blue), and Cam-CAN (orange) datasets. These distributions have had outliers removed. An example of the brainlife.io visualization for reference datasets can be found in Fig. S5. Data are presented as mean values ± SEM.

**Extended Data Fig. 7:**
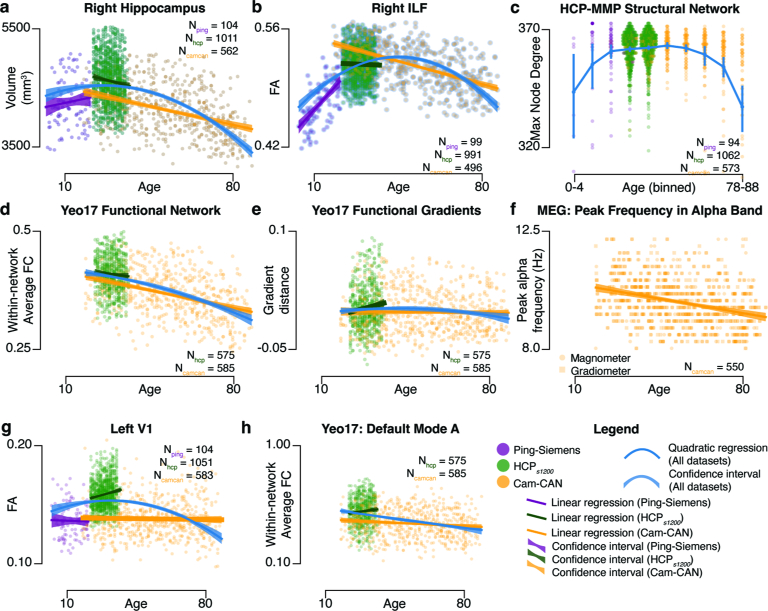
Lifelong brain maturation estimated across datasets. Relationship between subject age and **a**. Right hippocampal volume, **b**. Right inferior longitudinal fasciculus (ILF) fractional anisotropy (FA), **c**. maximum node degree of density network derived using the hcp-mmp atlas, d*. Within-network average functional connectivity (FC) derived using the Yeo17 atlas, e*. Functional gradient distance for visual resting state network derived from the Yeo17 atlas, and **f**. Peak frequency in the alpha band derived from magnetometer (squares) and gradiometers (circles) from MEG data. These analyses include subjects from the PING (purple), HCPs1200 (green), and Cam-CAN (yellow) datasets. Linear regressions were fit to each dataset, and a quadratic regression was fit to the entire dataset (blue). * All points in e, and f are presented. See Fig. 2a. Relationship between age of subject and **g**. Cortical fractional anisotropy (FA) of the left V1, **h**. Within-network average functional connectivity (FC) from the Yeo17 Default Mode - A network. These analyses include subjects from the PING (purple), HCPs1200 (green), and CAN (yellow) datasets. Linear regressions were fit to each dataset, and a quadratic regression was fit to the entire dataset (blue).

**Extended Data Fig. 8:**
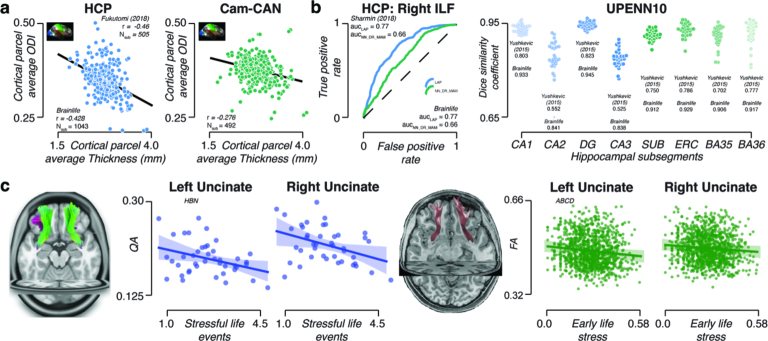
Replication of previous studies using brainlife.io. **a**. Average cortical hcp-mmp parcel thickness (Nstruc = 322) compared to parcel orientation dispersion index (ODI) from the NODDI model mapped to the cortical surface (inset) of the HCPS1200 dataset (Nsub = 1,043) and Cam-CAN (Nsub = 492) dataset compared to the parcel-average cortical thickness. **b**. Receiver operator curves (ROC) comparing the performance of segmentation of the Right ILF using two automated segmentation methods (LAP: blue, NN_DR_MAM: green) in a subset of the HCPS1200 dataset (Nsub = 15). Dice coefficients between manual and automated segmentation of the hippocampus using AHSS method in UPENN dataset. **c**. Stressful life events obtained from Negative Life Events Schedule (NLES) survey from Healthy Brain Network participants (Nsub = 42) compared to Uncinate-average normalized Quantitative Anisotropy (QA). Mean linear regression (blue line) fits and standard deviation (shaded blue). Early life stress was obtained from multiple surveys collected from ABCD participants (Nsub = 1,107) compared to Uncinate-average Fractional Anisotropy (FA). Linear regression (green line) fits the data with standard deviation (shaded green). See Fig. 2b,c.

We performed validation experiments to demonstrate cases where brainlife.io’s technology produces results consistent with best practices in the field. We used over 1,800 participants from three datasets: PING, HCP_s1200_ and Cam-CAN (Extended Data Figs. 3–8, Supplementary Results 4, Supplementary Fig. 4 and Supplementary Tables 3 and 4). Participants across all datasets spanned seven decades (that is, PING, 3–20 years; HCP_s1200_, 20–37 years and Cam-CAN, 18–88 years). Lifespan trajectories were plotted for multiple brain features (for example, brain region volume, white matter tract FAs, connectivity networks and MEG peak frequency; Fig. 2a and Extended Data Fig. 7) using brainlife.io’s Jupyter Notebooks. Inverted U-shaped lifespan trajectories were estimated, consistent with previous studies^15–17^ (Fig. 2a and Extended Data Fig. 7). The results generated using brainlife.io demonstrate that substantially different datasets can be collated to identify established brain’s lifespan trajectories (Supplementary Results 4).

We further evaluated the ability to replicate results and generalize findings. Apps were created to estimate cortical thickness and tissue orientation dispersion, orientation dispersion index (ODI) and analyze the HCP_s1200_ dataset. A negative relationship between cortical thickness and ODI was estimated (Fig. 2b and Extended Data Fig. 8; *r*_HCP-brainlife_ = −0.43 versus *r*_original_), replicating the original study (ODI; *r*_original_ = −0.46)^18^. The result was also generalized to the Cam-CAN dataset (Fig. 2b and Extended Data Fig. 8; *r*_Cam-CAN-brainlife_ = −0.28 versus *r*_original_). The association between life stressors and white matter organization of the uncinate fasciculus (*r* = −0.057) was a replication of Hason et al.^19^ using two independent datasets. The Negative Life Events Schedule (NLES) was correlated with quantitative anisotropy in the right- and left-hemisphere uncinate fasciculus (Fig. 2c and Extended Data Fig. 8; *r*_HBN_LEFT_ = −0.35, two-tailed *t*-test, *P* = 0.018; *r*_HBN_RIGHT_ = −0.39, two-tailed *t*-test *P* < 0.0156). Early Life Stress (a composite score of traumatic life events, environmental and neighborhood safety, and the family conflict subscale) was associated with the uncinate fasciculus FA (Fig. 2c and Extended Data Fig. 8; *r*_ABCD_LEFT_ = −0.12, *P* = 9.41 × 10^−5^; *r*_ABCD_RIGHT_ = −0.09, *P* = 0.0035). The results demonstrate the ability of brainlife.io services to detect meaningful associations in large, heterogeneous datasets (Supplementary Results 4).

Finally, we tested the ability of brainlife.io’s services to detect optic radiation white matter changes as a result of eye disease^20^. Individuals with Stargardt’s disease (deterioration initiated in the central retina) and choroideremia (deterioration initiated in peripheral retina) were compared to healthy controls. Stargardt’s FA was reduced in optic radiation fibers projecting to central V1 (not peripheral; Fig. 2d). Choroideremia’s FA was reduced in optic radiation fibers projecting to peripheral V1 (not central; Fig. 2d and Supplementary Results 4).

Our vision for brainlife.io is that of a trusted, interoperable and integrative platform connecting data archives and global communities of software developers, hardware providers and domain scientists (Supplementary Results 5 and Supplementary Table 5). The goal of brainlife.io is to facilitate research and education, accelerate brain understanding and lead to cures for brain diseases. To support this vision, brainlife.io connects trainees, researchers, developers and computing resource managers in high-, medium- and low-income countries via technology. The platform is registered on fairsharing.org, datacite.org and nitric.org, it is recommended by the International Neuroinformatics Coordinating Facility (https://incf.org/infrastructure/brainlife) and it can serve the US National Institutes of Health in the United States data deposition and sharing mandate^21,22^. A comprehensive overview of the platform and tutorials can be found at https://brainlife.io/docs. Videos provide tutorials and demonstrations at youtube.com/@brainlifeio. A slack channel supports communication and operations: https://brainlife.slack.com. Questions can be posted using the topic ‘brainlife’ on https://neurostars.org or GitHub issues (https://github.com/brainlife/brainlife/issues) can be added directly to the code repositories. A quarterly outreach newsletter is sent out to all users, and an X account (@brainlifeio) informs the wider community about critical events. The platform has already collected a growing community (Supplementary Results 3).

## Methods

### Data sources

Multiple openly available data sources were used for examining the validity, reliability and reproducibility of brainlife.io apps and for examining population distributions. All information regarding the specific image acquisitions, participant demographics and study-wide preprocessing can be found in the publications in refs. ^5–7,9,10,23–25^. Some data sources are currently unpublished. For these, the appropriate information is provided. Experiments were approved by the local institutional review boards (IRB) and only the personnel approved for a specific study accessed the data in private projects on brainlife.io.

### Validity, reliability, reproducibility, replicability, developmental trends and reference datasets

#### HCP (test–retest, s1200-release)

Data from these projects were used to assess the validity, reliability and reproducibility of the platform. They were used to assess the abilities of the platform to identify developmental trends in structural and functional measures, and they were used to generate reference datasets. For structural MRI (sMRI) data, the minimally preprocessed structural T1w and T2w images from the HCP from 1,066 participants from the s1200 and 44 participants from the test–retest releases were used^5^. Specifically, the 1.25 mm ‘acpc_dc_restored’ images generated from the Siemens 3 T MRI scanner were used for all analyses involving the HCP. For most examinations, the already-processed Freesurfer output from HCP was used. For diffusion MRI (dMRI) data, to assess the validity of preprocessing on brainlife.io, the unprocessed dMRI data from 44 participants from the HCP test dataset was used. For reliability and all remaining analyses, the minimally preprocessed dMRI images from 1,066 participants from the s1200 and 44 participants from the test–retest releases from the 3 T Siemens scanner were used. All processes incorporated the multi-shell acquisition data. For functional data (functional MRI (fMRI)), regarding validation, the unprocessed resting-state fMRI data from 44 participants from the HCP test dataset were compared to the minimally preprocessed blood oxygenation level dependent data provided by HCP. For reliability and all other analyses, the minimally preprocessed blood oxygenation level dependent data from 1,066 participants from the s1200 and 44 participants from the test–retest releases from the 3 T Siemens scanner were used.

#### The Cam-CAN

The data from this project were used to assess the validity, reliability and reproducibility of the platform, and to assess the abilities of the platform to identify developmental trends of structural and functional measures, and to generate reference datasets. For sMRI data, the unprocessed 1 mm isotropic structural T1w and T2w images from 652 participants from the Cam-CAN^6^ study were used. For dMRI data, the unprocessed 2 mm isotropic diffusion (dMRI) images from 652 participants from the Cam-CAN study were used. For fMRI data, the 3 × 3 × 4 mm^3^ unprocessed resting-state fMRI images from 652 participants from the Cam-CAN study were used. For electromagnetic data (MEG), the 1,000 Hz resting-state filtered and unfiltered datasets from 652 participants from the Cam-CAN study were used.

### Developmental trends and reference datasets

#### PING

The data from this project were used to assess the abilities of the platform to identify developmental trends of structural measures and to generate reference datasets. For sMRI data, the unprocessed 1.2 × 1.0 × 1.0 mm^3^ structural T1w and the 1.0 mm isotropic T2w images from 110 participants from the PING^10^ study were used. For dMRI data, the unprocessed 2 mm isotropic diffusion (dMRI) images from 110 participants from the PING study were used.

### Replicability datasets

#### ABCD

For sMRI data, the unprocessed 1 mm isotropic structural T1w and T2w images from a subset of 1,877 participants from the ABCD (release-2.0.0) study were used. For dMRI data, the unprocessed 1.77 mm isotropic diffusion (dMRI) images from a subset of 1,877 participants from the ABCD (release-2.0.0) study were used^7,26^. A single diffusion gradient shell was used for these experiments (*b* = 3,000 s ms^−^^2^). Research was approved by the University of Arkansas IRB (no. 2209425822).

#### HBN

The data from this project were used to assess the abilities of the platform to replicate previously published findings via the assessment of the relationship between microstructural measures mapped to segmented uncinate fasciculi and self-reported early life stressors. Research was approved by the University of Pittsburgh IRB (no. PRO17060350). For sMRI data, the 0.8 mm isotropic structural T1w images from 42 participants from the HBN study^9^ were used. For dMRI data, the unprocessed 1.8 mm isotropic diffusion (dMRI) images from 42 participants from the CitiGroup Cornell Brain Imaging Center site of the HBN study were used. Research was approved by the University of Pittsburgh IRB (no. PRO17060350).

#### UPENN-PMC

The University of Pennsylvania, Penn Memory Center (UPENN-PMC) data from this project were used to assess the abilities of the platform to replicate previously published findings via the assessment of the performance of an automated hippocampal segmentation algorithm. Secondary data analyses were conducted under IRB exemption at Indiana University. For sMRI data, the T1w and T2w data were provided within the Automated Segmentation of Hippocampal Subfields Automated Segmentation of Hippocampal Subfields atlas^27^.

### Clinical-identification datasets

#### Indiana University Acute Concussion dataset

The data from this project were used to assess the abilities of the platform to identify clinical populations via the mapping of microstructural measures to the cortical surface. Neuroimaging was performed at the Indiana University Imaging Research Facility, housed within the Department of Psychological and Brain Sciences with a 3 T Siemens Prisma whole-body MRI using a 64-channel head coil. Within this study, nine concussed athletes and 20 healthy athletes were included. Research approved by Indiana University (IRB 906000405). For sMRI data, high-resolution T1-weighted structural volumes were acquired using an MPRAGE sequence: TI = 900 ms, TE = 2.7 ms, TR = 1,800 ms, flip angle 9°, with 192 sagittal slices of 1.0 mm thickness, a field of view of 256 × 256 mm^2^ and an isometric voxel size of 1.0 mm^3^ (where TI, TE and TR refer to inversion time, echo time and repetition time, respectively). The total acquisition time was 4 min and 34 s. High-resolution T2-weighted structural volumes were also acquired: TE = 564 ms, TR = 3,200 ms, flip angle 120°, with 192 sagittal slices, a field of view of 240 × 256 mm^2^ and an isometric voxel size of 1.0 mm^3^. Total acquisition time was 4 min and 30 s. Diffusion data (dMRI) were collected using single-shot spin-echo simultaneous multi-slice (SMS) echo-planar imaging (transverse orientation, TE = 92.00 ms, TR = 3,820 ms, flip angle 78°, isotropic 1.5 mm^3^ resolution; FOV = LR 228 × 228 × 144 mm^3^; acquisition matrix MxP 138 × 138. SMS acceleration factor 4). This sequence was collected twice, one in the anterior-posterior fold-over direction and the other in the posterior-anterior (PA) fold-over direction, with the same diffusion gradient strengths and the number of diffusion directions: 30 diffusion directions at *b* = 1,000 s mm^−^^2^, 60 diffusion directions at *b* = 1,750 s mm^2^, 90 diffusion directions at *b* = 2,500 s mm^2^ and 19 *b* = 0 s mm^2^ volumes. The total acquisition time for both sets of dMRI sequences was 25 min and 58 s.

#### Oxford University Choroideremia & Stargardt’s Disease Dataset

The data from this project were used to assess the abilities of the platform to identify clinical populations via mapping retinal-layer thickness via optical coherence tomography and mapping of microstructural measures along optic radiation bundles segmented using visual field information (eccentricity). Neuroimaging was performed at the Wellcome Centre for Integrative Neuroimaging, Oxford with the Siemens 3 T scanner. Research was approved by the UK Health Regulatory Authority reference 17/LO/1540. For sMRI data, high-resolution T1-weighted anatomical volumes were acquired using an MPRAGE sequence: TI = 904 ms, TE = 3.97 ms, TR = 1,900 ms, flip angle 8°, with 192 sagittal slices of 1.0 mm thickness, a field of view of 174 × 192 × 192 mm^3^ and an isometric voxel size of 1.0 mm^3^. The total acquisition time was 5 min and 31 s. Diffusion data (dMRI) were collected using echo-planar imaging (transverse orientation, TE = 92.00 ms, TR = 3,600 ms, flip angle 78°, 2.019 × 2.019 × 2.0 mm^3^ resolution; FOV = 210 × 220 × 158 mm^3^; acquisition matrix MxP = 210 × 210, SMS acceleration factor 3). This sequence was collected twice, one in the anterior-posterior fold-over direction and the other in the PA fold-over direction. The PA fold-over scan contained six diffusion directions, three at *b* = 0 s mm^2^ and three at *b* = 2,000 s mm^−^^2^, and was used primarily for susceptibility-weighted corrections. The anterior-posterior fold-over scan contained 105 diffusion directions, five at *b* = 0 mm s^−^^2^, 51 at *b* = 1,000 mm s^−^^2^ and 49 at *b* = 2,000 mm s^−^^2^. The total acquisition time for both sets of dMRI sequences was 7 min and 8 s.

### General processing pipelines

#### Structural processing

For the ABCD, Cam-CAN, Oxford University Choroideremia & Stargardt’s Disease Dataset, and the Indiana University Acute Concussion datasets, the structural T1w and T2w (sMRI) images (if available) were preprocessed, including bias correction and alignment to the anterior commissure-posterior commissure plane, using the brainlife.io apps A273 (10.25663/brainlife.app.273) and A350 (10.25663/brainlife.app.350), respectively. For PING data, no bias correction was performed but alignment to the anterior commissure-posterior commissure plane was performed using A99 (10.25663/brainlife.app.99) and A116 (10.25663/brainlife.app.116) for T1w and T2w data, respectively. For HCP data, this data was already provided. The structural T_1_-weighted images for each participant and dataset were then segmented into different tissue types using functionality provided by MRTrix3 (ref. ^28^) implemented as A239 (10.25663/brainlife.app.239). For a subset of datasets, this was performed within the diffusion tractography generation step using A319 (10.25663/brainlife.app.319). The gray- and white-matter interface mask was subsequently used as a seed mask for white matter tractography. The processed structural T1w and T2w images were then used for segmentation and surface generation using the recon-all function from Freesurfer^29^ (A0; 10.25663/brainlife.app.0). Following Freesurfer, representations of the cortical ‘midthickness’ surface were computed by spatially averaging the coordinates of the pial and white matter surfaces generated by Freesurfer using the wb_command -surface-cortex-layer function provided by Workbench command for the HCP_TR_, HCP_s1200_, ABCD, Cam-CAN, PING and Indiana University Acute Concussion datasets. These surfaces were used for cortical tissue mapping analyses. Following Freesurfer and midthickness-surface generation, the 180 multimodal cortical nodes (hcp-mmp) atlas and the Yeo 17 (yeo17) atlas were mapped to the Freesurfer segmentation of each participant implemented as brainlife.io app A23 (10.25663/brainlife.app.23). These parcellations were used for subsequent cortical, subcortical and network analyses. In addition, measures for cortical thickness, surface area, volume and summaries of diffusion models of microstructure were estimated using A383 (10.25663/brainlife.app.383) and A389 (10.25663/brainlife.app.389). To estimate population receptive fields and visual field eccentricity properties in the cortical surface in the Oxford University Choroideremia & Stargardt’s Disease Dataset, the automated mapping algorithm developed by refs. ^30,31^ was implemented using A187 (10.25663/brainlife.app.187). To segment thalamic nuclei for optic radiation tracking, the automated thalamic nuclei segmentation algorithm provided by Freesurfer^28^ was implemented as A222 (10.25663/brainlife.app.222). Finally, visual regions of interest (ROI) binned by eccentricity were then generated using AFNI software^32^ functions implemented in A414 (10.25663/brainlife.app.414). To assess the replicability capabilities of the platform, an automated hippocampal nuclei segmentation app (A262; 10.25663/brainlife.app.262) was used to segment hippocampal subfields from participants within the UPENN-PMC dataset provided within the Automated Segmentation of Hippocampal Subfields atlas.

### dMRI processing

#### Preprocessing and model fitting

For most of the analyses involving the HCP dataset, the minimally preprocessed dMRI images were used and thus no further preprocessing was performed. However, to assess the validity of the preprocessing pipeline, the unprocessed dMRI data from the HCP test dataset and dMRI images were preprocessed following the protocol outlined in ref. ^33^ using A68 (10.25663/brainlife.app.68). The same app was also used for preprocessing the dMRI images for the ABCD, Cam-CAN, PING, Oxford University Choroideremia & Stargardt’s Disease Dataset, the Indiana University Acute Concussion and HBN datasets. Specifically, dMRI images were denoised and cleaned from Gibbs ringing using functionality provided by MRTrix3 before being corrected for susceptibility, motion and eddy distortions and artifacts using FSL’s topup and eddy functions^34,35^. Eddy-current and motion correction was applied via the eddy_cuda8.0 with the replacement of outlier slices (that is, repol) command provided by FSL^36–39^. Following these corrections, MRTrix3’s dwigradcheck functionality was used to check and correct for potential misaligned gradient vectors following topup and eddy^40^. Next, dMRI images were debiased using ANT’s n4 functionality^41^ and the background noise was cleaned using MrTrix3.0’s dwidenoise functionality^42^. Finally, the preprocessed dMRI images were registered to the structural (T1w) image using FSL’s epi_reg functionality^43–45^. Following preprocessing, brain masks for dMRI data using bet from FSL were implemented as A163 (10.25663/brainlife.app.163).

#### DTI, NODDI and *q*-sampling model fitting

Following preprocessing, the diffusion tensor imaging (DTI) model^46^ and the neurite orientation dispersion and density imaging (NODDI)^47,48^ models were subsequently fit to the preprocessed dMRI images for each participant using either A319 (10.25663/brainlife.app.319) or A292 (10.25663/brainlife.app.292) for DTI model fitting and A365 (10.25663/brainlife.app.365) for NODDI fitting. Note, the NODDI model was only fit on the HCP, Cam-CAN, Oxford University Choroideremia & Stargardt’s Disease Dataset and the Indiana University Acute Concussion datasets. For those datasets, the NODDI model was fit using an intrinsic free diffusivity parameter (*d*_∥_*)* of 1.7 × 10^−3^ mm^2^ s^−1^ for white matter tract and network analyses, and a *d*_∥_ of 1.1 × 10^−3^ mm^2^ s^−1^ for cortical tissue mapping analyses, using AMICO’s implementation^48^ as A365 (10.25663/brainlife.app.365). The constrained spherical deconvolution^49^ model was then fit to the preprocessed dMRI data for each run across four spherical harmonic orders (that is, *L*_max_) parameters (2, 4, 6, 8) using functionality provided by MRTrix3 implemented as brainlife.io app A238 (10.25663/brainlife.app.238). For the PING datasets, the constrained spherical deconvolution model was fit using the same code found in A238 (10.25663/brainlife.app.238), but performed using the tractography app A319 (10.25663/brainlife.app.319). For the HBN dataset, the isotropic spin distribution function was obtained by reconstructing the diffusion MRI data with the generalized *q*-sampling imaging method^50^ using functionality provided by DSI-Studio^51^ (A423; 10.25663/brainlife.app.423). Quantitative anisotropy was then estimated from the isotropic spin distribution function.

#### Tractography

Following model fitting, the fiber orientation distribution functions for *L*_max_ = 6 and *L*_max_ = 8 were subsequently used to guide anatomically constrained probabilistic tractography^52^ using functions provided by MRTrix3 implemented as brainlife.io app A297 (10.25663/brainlife.app.297) or A319 (10.25663/brainlife.app.319). For the HCP_TR_, HCP_s1200_ and Oxford University Choroideremia & Stargardt’s Disease datasets, *L*_max_ = 8 was used. For the ABCD and Cam-CAN datasets, *L*_max_ = 6 was used. For the HCP, ABCD and Cam-CAN, datasets, a total of 3 million streamlines were generated. For all datasets, a step size of 0.2 mm was implemented. For the HCP_TR_, HCP_s1200_, ABCD and Cam-CAN datasets, minimum and maximum lengths of streamlines were set at 25 and 250 mm, respectively, and a maximum angle of curvature of 35° was used. For the PING dataset, minimum and maximum lengths of streamlines were set at 20 and 220 mm, respectively, and a maximum angle of curvature of 35° was used.

#### Whiter matter segmentation and cleaning

Following tractography, 61 major white matter tracts were segmented for each run using a customized version of the white matter query language^53^ implemented as brainlife.io app A188 (10.25663/brainlife.app.188). Outlier streamlines were subsequently removed using functionality provided by Vistasoft and implemented as brainlife.io app A195 (10.25663/brainlife.app.195). Following cleaning, tract profiles with 200 nodes were generated for all DTI and NODDI measures across the 61 tracts for each participant and test–retest condition using functionality provided by Vistasoft and implemented as A361 (10.25663/brainlife.app.361). Macrostructural statistics, including average tract length, tract volume and streamline count were computed using functionality provided by Vistasoft implemented as A189 (10.25663/brainlife.app.189). Microstructural and macrostructural statistics were then compiled into a single data frame using A397 (10.25663/brainlife.app.397).

#### Segmentation of the optic radiation

To generate optic radiations segmented by estimates of visual field eccentricity in the Oxford University Choroideremia & Stargardt’s Disease Dataset, ConTrack^54^ tracking was implemented as A252 (10.25663/brainlife.app.252). Then, 500,000 sample streamlines were generated using a step size of 1 mm. Samples were then pruned using inclusion and exclusion waypoint ROI following methodologies outlined in refs. ^19,55^.

#### Segmentation of uncinate fasciculus

To assess the relationship between uncinate tract-average quantitative anisotropy, fractional anisotropy (FA) and early life stressors within two independent datasets (HBN, ABCD), the tract-average quantitative anisotropy for the left and right uncinate were computed from 42 participants from the HBN and the tract-average FA were computed from 1,107 participants from the ABCD dataset. For the HBN dataset, a full tractography segmentation pipeline was used to preprocess the dMRI data and segment the uncinate fasciculus using A423 (10.25663/brainlife.app.423). Automatic fiber tracking was then performed to segment the uncinate fasciculus using default parameters and templates from a population tractography atlas from the HCP^56^. A threshold of 16 mm as the maximum allowed threshold for the shortest streamline distance was then applied to remove spurious streamlines. The whole tract-average quantitative anisotropy was then estimated. To probe stress exposure within the HBN dataset, we used the NLES, a 22-item questionnaire in which participants were asked about the occurrence of different stressful life events. The tractography pipeline for the ABCD dataset has been described previously. The average FA for the left and right uncinate were estimated using procedures described previously, and then compared to the participant’s life stressors behavioral measures by fitting a linear regression to the data.

#### Structural networks

Following tract segmentation, structural networks were generated using the multimodal 180 cortical node atlas and the tractograms for each participant using MRTrix3’s tck2connectome (ref. ^57^) functionality implemented as A395 (10.25663/brainlife.app.395). Connectomes were generated by computing the number of streamlines intersecting each ROI pairing in the 180 cortical node parcellation. Multiple adjacency matrices were generated, including count, density (that is, the count divided by the node volume of the ROI pairs), length, length density (that is length divided by the volume of the ROI pairs) and average and average density axial diffusivity, fractional anisotropy, mean diffusivity, radial diffusivity, neurite density index, orientation dispersion index and isotropic volume fraction. Density matrices were generated using the -invnodevol option^58^. For non-count measures (length, axial diffusivity, fractional anisotropy, mean diffusivity, radial diffusivity, neurite density index, orientation dispersion index, isotropic volume fraction), the average measure across all streamlines connecting and ROI pair was computed using MRTrix3’s tck2scale functionality using the -precise option^59^ and the -scale_file option in tck2connectome. These matrices can be thought of as the ‘average measure’ adjacency matrices. These files were output as the ‘raw’ datatype and were converted to a conmat datatype using A393 (10.25663/brainlife.app.393). Connectivity matrices were then converted into the ‘network’ datatype using functionality from Python functionality implemented as A335 (10.25663/brainlife.app.335).

#### Cortical and subcortical diffusion and morphometry mapping

For the PING, HCP_TR_, HCP_s1200_, Cam-CAN and Indiana University Acute Concussion datasets, DTI and NODDI (if available) measures were mapped to each participant’s cortical white matter parcels following methods found in Fukutomi and colleagues^18^ using functions provided by Connectome Workbench^60^ implemented as brainlife.io app A379 (10.25663/brainlife.app.379). A Gaussian smoothing kernel (full-width at half-maximum ~4 mm, *σ* = 5/3 mm) was applied along the axis normal to the midthickness surface, and DTI and NODDI measures were mapped using the wb_command -volume-to-surface-mapping function. Freesurfer was used to map the average DTI and NODDI measures within each parcel using functionality from Connectome Workbench using A389 (10.25663/brainlife.app.389) and A483 (10.25663/brainlife.app.483). Measures of volume, surface area and cortical thickness for each cortical parcel were computed using Freesurfer and A464 (10.25663/brainlife.app.464). Freesurfer was also used to generate parcel-average DTI and NODDI measures for the subcortical segmentation (aseg) from Freesurfer using A383 (10.25663/brainlife.app.383). Measures of volume for each subcortical parcel were computed using Freesurfer and A272 (10.25663/brainlife.app.272).

### rs-fMRI preprocessing and functional connectivity matrix generation

For the HCP_TR_ and Cam-CAN datasets, unprocessed resting-state functional MRI (rs-fMRI) datasets were preprocessed using fMRIPrep implemented as A160 (10.25663/brainlife.app.160). Briefly, fMRIPrep does the following preprocessing steps. First, individual images are aligned to a reference image for motion estimation and correction using mcflirt from FSL. Next, slice timing correction is performed in which all slices are realigned in time to the middle of each relaxation time using 3dTShift from AFNI. Spatial distortions are then corrected using field map estimations. Finally, the fMRI data is aligned to the structural T1w image for each participant. Default parameters provided by fMRIPrep were used. For a subset of analyses involving the HCP test and retest datasets, the preprocessed rs-fMRI datasets provided by the HCP consortium were used. Following preprocessing via fMRIPrep for the volume data, connectivity matrices were generated using the Yeo17 parcellation and A369 (10.25663/brainlife.app.369) and A532 (10.25663/brainlife.app.532). Within-network functional connectivity for the 17 canonical resting-state networks was computed by computing the average functional connectivity values within all of the nodes belonging to a single network. These estimates were used for subsequent analyses.

### rs-fMRI gradient processing

For the HCP_TR_ and Cam-CAN datasets, unprocessed rs-fMRI data from the HCP Test and Cam-CAN datasets were preprocessed using fMRIPrep implemented as A160 (10.25663/brainlife.app.160). Within this app, the same preprocessing steps are undertaken as in A160 (10.25663/brainlife.app.160), except for an additional volume-to-surface mapping using mri_vol2surf from Freesurfer. The surface-based outputs were then used to compute gradients following methodologies outlined in ref. ^61^ for each participant in the HCP_s1200_, HCP_TR_ and Cam-CAN datasets using A574 (10.25663/brainlife.app.574) using diffusion embedding^62^ and functions provided by BrainSpace^63^. More specifically, connectivity matrices were computed from surface vertex values within each node of the Schaffer 1,000 parcellation^64^. Cosine similarity was then computed to create an affinity matrix to capture inter-area similarity. Dimensionality reduction is then used to identify the primary gradients. A normalized-angle kernel was used to create the affinity matrix, from which two primary components were identified. Gradients were then aligned across all participants using a Procrustes alignment and joined embedding procedure^61^. Values from the primary gradient and the cosine distance used to generate the affinity matrices were used for subsequent analyses.

### MEG processing

For some analyses, raw resting-state-MEG time series data from the Cam-CAN dataset was filtered using a Maxwell filter implemented as A476 (10.25663/brainlife.app.476) and median split using A529 (10.25663/brainlife.app.529). For the remainder of the analyses, filtered data provided by the Cam-CAN dataset was used. For all MEG data, power-spectrum density profiles (PSD) were estimated using functionality provided by MNE-Python^28,65^ implemented as A530 (10.25663/brainlife.app.530). Following PSD estimation, peak alpha frequency was estimated using A531 (10.25663/brainlife.app.531). Finally, PSD profiles were averaged across all nodes within each of the canonical lobes (frontal, parietal, occipital, temporal) using A599 (10.25663/brainlife.app.599). Measures of PSD and peak alpha frequency were used for all subsequent analyses.

### Reporting summary

Further information on research design is available in the Nature Portfolio Reporting Summary linked to this article.

## Online content

Any methods, additional references, Nature Portfolio reporting summaries, source data, extended data, supplementary information, acknowledgements, peer review information; details of author contributions and competing interests; and statements of data and code availability are available at 10.1038/s41592-024-02237-2.

### Supplementary information

Supplementary Information Supplementary Figs. 1–5, Tables 1–5 and Results 1–5.

Reporting Summary

## Figures and Tables

**Extended Data Table 1:**
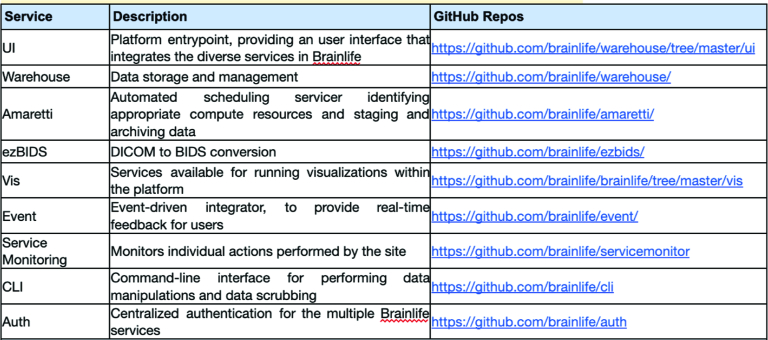
Platform microservices

Table with list of all platform services, name, scope, service URL (pointer to brainlife page if available as direct URL) and github URL for code.

## Data Availability

All data derived and described in this paper are made available via the brainlife.io platform as ‘Publications’. User data agreements are required for some projects, like data from the HCP, Cam-CAN, PING, ABCD and HBN datasets. The Indiana University Acute Concussion and Oxford University Choroideremia & Stargardt’s Disease Datasets are part of ongoing research projects and will be made available at a later stage. All other datasets are made freely available via the brainlife.io platform. See Supplementary Table 6 for the brainlife.io/pubs.

## Code availability

As part of the article, we are describing a total of nine platform components. All components are made publicly available and open source under MIT License. All the software for the platform components is listed in Supplementary Table 1. In addition, we share the code used for the statistical analyses as Jupyter Notebooks (Supplementary Table 2). Finally, the Apps used and tested in this article are listed in Supplementary Table 3.
