# Author Correction: brainlife.io: a decentralized and open-source cloud platform to support neuroscience research

**DOI:** 10.1038/s41592-024-02296-5

**Published:** 2024-05-07

**Authors:** Soichi Hayashi, Bradley A. Caron, Anibal Sólon Heinsfeld, Sophia Vinci-Booher, Brent McPherson, Daniel N. Bullock, Giulia Bertò, Guiomar Niso, Sandra Hanekamp, Daniel Levitas, Kimberly Ray, Anne MacKenzie, Paolo Avesani, Lindsey Kitchell, Josiah K. Leong, Filipi Nascimento-Silva, Serge Koudoro, Hanna Willis, Jasleen K. Jolly, Derek Pisner, Taylor R. Zuidema, Jan W. Kurzawski, Kyriaki Mikellidou, Aurore Bussalb, Maximilien Chaumon, Nathalie George, Christopher Rorden, Conner Victory, Dheeraj Bhatia, Dogu Baran Aydogan, Fang-Cheng F. Yeh, Franco Delogu, Javier Guaje, Jelle Veraart, Jeremy Fischer, Joshua Faskowitz, Ricardo Fabrega, David Hunt, Shawn McKee, Shawn T. Brown, Stephanie Heyman, Vittorio Iacovella, Amanda F. Mejia, Daniele Marinazzo, R. Cameron Craddock, Emanuale Olivetti, Jamie L. Hanson, Eleftherios Garyfallidis, Dan Stanzione, James Carson, Robert Henschel, David Y. Hancock, Craig A. Stewart, David Schnyer, Damian O. Eke, Russell A. Poldrack, Steffen Bollmann, Ashley Stewart, Holly Bridge, Ilaria Sani, Winrich A. Freiwald, Aina Puce, Nicholas L. Port, Franco Pestilli

**Affiliations:** 1 Indiana University, Bloomington, IN USA; 2 The University of Texas, Austin, TX USA; 3 Vanderbilt University, Nashville, TN USA; 4 McGill University, Montréal, Quebec Canada; 5 Cajal Institute, CSIC, Madrid, Spain; 6 Fondazione Bruno Kessler, Trento, Italy; 7 Applied Physics Laboratory, Johns Hopkins University, Laurel, MD USA; 8 University of Arkansas, Fayetteville, AR USA; 9 University of Oxford, Headington, Oxford, UK; 10 Anglia Ruskin University, Cambridge, UK; 11 New York University, New York, NY USA; 12 University of Limassol, Nicosia, Cyprus; 13 University of Cyprus, Nicosia, Cyprus; 14 Institut du Cerveau, CNRS, Sorbonne Université, Paris, France; 15 University of South Carolina, Columbia, SC USA; 16 Lawrence Technological University, Southfield, MI USA; 17 University of Eastern Finland, Kuopio, Finland; 18 Aalto University School of Science, Espoo, Finland; 19 University of Pittsburgh, Pittsburgh, PA USA; 20 University of Michigan, Ann Arbor, MI USA; 21 Hewlett-Packard Enterprise, Pittsburgh, PA USA; 22 SHEGEL, Massul, Luxembourg; 23 University of Trento, Rovereto, Italy; 24 University of Ghent, Ghent, Belgium; 25 University of Nottingham, Nottingham, UK; 26 Stanford University, Stanford, CA USA; 27 University of Queensland, St Lucia, Queensland Australia; 28 The Rockefeller University, New York, NY USA; 29 University of Geneva, Geneva, Switzerland

**Keywords:** Cognitive neuroscience, Computational neuroscience, Translational research, Technology, Databases

Correction to: *Nature Methods* 10.1038/s41592-024-02237-2, published online 11 April 2024

In the version of the article initially published, Steffen Bollmann’s surname was misspelled (as Bollman) and has now been corrected in the HTML and PDF versions of the article.

