# Supplementary material for: brainlife.io: a decentralized and open-source cloud platform to support neuroscience research
Source: Nat Methods. 2024 Apr 11;21(5):809–13. doi: 10.1038/s41592-024-02237-2 (PMC11093740; doi:10.1038/s41592-024-02237-2)
Supplement: Supplementary file 1 — Supplementary Figs. 1–5, Tables 1–5 and Results 1–5. [file 41592_2024_2237_MOESM1_ESM.pdf]

nature methods

Brief Communication https://doi.org/10.1038/s41592-024-02237-2

brainlife.io: a decentralized and open-source cloud platform to support neuroscience research

In the format provided by the authors and unedited

Supplementary Results **1**
Platform architecture **1**
Supplementary Figure 1. Brainlife App Github template. 6
Developing processing Apps for the platform 6
Using the platform **7**
Supplementary Figure 2. Brainlife project landing page. 7
End-to-end reproducible scientific workflow **8**
Platform utilization **9**
Supplementary Figure 3. Brainlife.io processing is not error-proof. 10
Platform testing **10**
Supplementary Figure 4. Overview of data used for the study. 11
Quality control at scale 13
Supplementary Figure 5. brainlife.io interface can visualize reference datasets. 14
Platform utility 14
Replication and generalization 15
Detecting disease 16
Public services for promoting transparency and data gravity in neuroscience research. **17**
Current limitations and future development to improve platform usability and data gravity **18**
Supplementary Table 1: Videos demonstrating use of platform. 20
Supplementary Table 2: Jupyter notebooks for analyses performed. 20
Supplementary Table 3: Preprocessing Apps used for the experiments. 22
Supplementary Table 4. Validity and reliability correlation tables. 24
Supplementary Table 5: Resources for data storage, archiving, and computational analysis. 25
Supplementary Table 6: Processed dataset published as part of this article. 27
References **28**

Supplementary Results

Supplementary Results 1: Platform architecture

The goal of the platform is to advance the democratization of big data neuroscience by lowering the barriers of entry to multimodal data analysis, network neuroscience, and large-scale analysis, all opportunities historically limited to a paucity of highly-skilled, high-profile research teams.[1–8] The platform supports a rigorous and transparent scientific process spanning the research data lifecycle from after data collection to sharing[9] and automatically tracks complex sequences of interactions between researchers, Apps, analysis notebooks, and data objects to support reproducibility. The platform's geographically distributed computing and storage systems are securely hosted by national supercomputing centers and funded by a combination of institutional, national, and international awards (see **Extended Data Figure 1a,b**). As of this time, the Texas Advanced Computing Center, Indiana University Pervasive Technology Institute, Pittsburgh Supercomputing Center, San Diego Supercomputing Center, and the University of Michigan Advanced Research Computing Technology Services have supported the project. The distributed platform is connected with and depends on other major infrastructure and software projects such as OpenNeuro.org, osris.org, DataLad.org, BIDS, Freesurfer, FSL, nibabel, dipy.org, repronim.org, DSI-Studio, jetstream-cloud.org, frontera-portal.tacc.utexas.edu, access-ci.org, and INCF.org.

brainlife.io is a composition of microservices, including authentication, preprocessing, warehousing, event handling, and auditing. Microservices are handled by a meta-orchestration workflow system, Amaretti (**Extended**

Data Figure 1c, and **Extended Data Table 1**). Amaretti can deploy computational jobs on high-performance compute clusters and cloud systems. Both jobs needed for platform operations and data analysis are handled by Amaretti. Amaretti is central to *brainlife.io*'s opportunistic computing approach, i.e., the ability to use donated storage or computing resources. Amaretti allows secure access to either clouds or supercomputers managing platform task scheduling, data transfer, and job submission and monitoring. Amaretti's core concepts are data- and resource awareness, i.e., data products or compute resources are specified as objects that the platform has explicit awareness of (e.g., the platform can dock datatypes, or compute resources; **Extended Data Figure 1d,e**). For example, users and resource managers can register a computing resource, making it available via *brainlife.io* either privately (to a specified set of users) or widely (to the entire platform users base). A variety of resource architectures and job submission systems have been tested and docked using Amaretti so far, including SLURM, PBS, OSG Engine, and CONDOR. Currently, Amaretti is hosted by a public cloud [10,11] and connected to major data centers (via access-ci.org) and commercial clouds.

Data processing on *brainlife.io* utilizes an object-oriented service model, based on micro workflows. Apps and datatypes work together to allow smart docking and awareness (**Extended Data Figure 1d,e**). Apps are modular, composable processing units comprising either full pipelines [12–32] or small steps within a larger data-processing workflow. Apps are written in a variety of languages following a lightweight specification (github.com/brainlife/abcd-spec) and using containerization technology [33,34]. Containerization allows deployment on various compute resource architectures (hub.docker.com/u/brainlife). Apps code is hosted on github.com. Code must be first registered on *brainlife.io* in order to become an App. An App registration process guides developers to map both input and output data objects to *brainlife.io* datatypes via a graphical interface. For security reasons, platform administrator approval is required to allow Apps on compute resources. A DOI [35–37] is issued for registered Apps to support scientific transparency and credit assignment to developers [5,38–47]. App specification requires developers to provide an informative readme file on GitHub with proper citations to software and funding used for the App (**Supplementary Figure 1**). After registration, platform users can access Apps via a graphical (GUI) or command line interface (CLI). Apps can run on multiple resources, and Amaretti has methods for matching Apps to resources based on criteria such as geolocation, performance profiles, and resource queue length.

Apps on *brainlife.io* are data-aware and can automatically identify datasets, dock them or send them elsewhere for processing. This is because data objects are stored using predefined formats —datatypes. Datatypes allow App concatenation and automated pipelining (**Figure 1d; Supplementary Figure 1**; brainlife.io/datatypes). Datatypes comprise collections of files and folders organized into *.tar* archives to limit the number of inodes needed for storage. A platform-side datatype validation service (github.com/brainlife/?q=validator-) assures that datatypes comply with their definition. Data are physically stored using S3-like storage buckets organized following the pattern: `<s3bucketName>/<projectID>/<datasetID>.tar` Buckets can live in multiple geolocations, so as to help with international requirements [9]. Datatypes comply with BIDS[48] (if the standard is defined for the data objects).

Data management is centered around Projects and supported by a databasing and warehousing system (github.com/brainlife/warehouse). Projects are the "one-stop-shop" for data management, processing, analysis, visualization, and publication (**Supplementary Figure 2**). Projects are created independently by users and are private by default, but can be made public within the *brainlife.io* platform. Projects provide stratified access control mechanisms, and data user agreements can be added to the landing page (see **Supplementary Table 1**). A project can be populated with data using several options (**Supplementary Figure 2**). Major archives and data repositories are docked by brainlife.io[49] (see **Extended Data Figure 1d,e**). Noticeable examples are OpenNeuro.org [50], and the Nathan-Kline data-sharing project [51–53]. Datasets can be seamlessly imported into *brainlife.io* Projects via the portal brainlife.io/datasets (see **Supplementary Table 1**). MRI, EEG, and MEG files (e.g., DICOM, .fif, .ctf) can also be uploaded directly using either a GUI (**Supplementary Table 1**) or CLI (**Supplementary Table 1**). A DICOM to BIDS conversion service has also been developed for MRI data standardization and importing into Projects (brainlife.io/ezbids; see **Extended Data Table 1** and **Supplementary Table 1**). Community-developed data visualizers are served by *brainlife.io* to support quality control (see **Extended Data Table 1**). Six new data visualizers have been developed and released as part of the project (**Extended Data Table 1** and **Supplementary Table 1**).

The data workflow in *brainlife.io* reduces the complexity of the neuroimaging processing pipeline into two steps akin to the MapReduce algorithm [54]. An initial *map step* preprocesses data objects asynchronously, is parallel using Apps, so as to extract features of interest, such as functional or white matter maps, or time series data (**Figure 1d**). During the *map step*, datatypes and Apps are synchronized and moved across available compute resources automatically, as optimized by Amaretti. Apps process data objects automatically and in parallel across study participants in a Project. A dedicated web interface exists to explore sequences of Apps and optimize the parameters for each data set (**Supplementary Table 1**). In addition, App sequences can be composed using a Pipeline builder interface (**Supplementary Table 1**).

The *map step* is followed by a *reduce step*. Features extracted using Apps are synchronized, brought together, and made available to Jupyter notebooks[55,56] for statistical analysis and to generate figures for scientific articles (all figures in the following sections of this paper are available in Jupyter notebooks, see **Supplementary Table 2**). App developers can identify datatypes as "statistical features", which are made accessible via Jupyter Lab interfaces hosted inside a Project (**Figure 1d** right, and **Supplementary Table 1**). The statistical features are automatically organized by *brainlife.io* into *Tidy data* formats [57] (*.tsv* and *.json*) and can be exported using the *pybrainlife* Python module (https://pypi.org/project/pybrainlife/). Jupyter Lab records are tracked for reproducibility and allow data analysis in R, Python, or Octave [55,56].

Currently, brainlife.io allows users to perform analyses requiring brain data and associated phenotypic or behavioral data in two primary ways; using BIDS modality-specific files or BIDS modality-agnostic files. Modality-specific files: Some of the existing Apps can take as input BIDS Modality-Specific Files, such as the Task event.tsv files, as described by the BIDS standard. These Apps can be used, for example, to perform first-level analysis combining fMRI, MEG, and EEG brain data and modality-specific files, such as Task events.tsv. In the future, other Apps can be developed to allow the introduction of new analyses and modality-specific files as they become available in the BIDS standard. Modality agnostic files: Alternatively, group-level data stored in BIDS Modality Agnostic files such as the participants.tsv, or phenotypic and assessment data (see here for a reference to the current BIDS specification for these files) can be imported into the Jupyter Notebooks hosted by brainlife.io to implement advanced statistical analysis, second level analysis and by combining advanced data features extracted using Apps with behavioral, or phenotypic and assessment data.

The full data workflow (from import to preprocessing to analysis) makes possible the unification of large volumes of diverse neuroimaging datatypes into simpler sets of features organized into *Tidy data* structures [57]. The platform provides a variety of methods to visualize data, which aids in performing quality assurance, identifying mistakes, and repeating the processing when needed. Community-developed visualizers are served on the cloud side using docker containers (see **Extended Data Table 1**), and six new web visualizers have been developed (**Extended Data Table 1** and **Supplementary Table 1**).

The ABCD specification and brainlife.io Apps. The Application for Big Computational Data (ABCD; github.com/brainlife/abcd-spec) is a lightweight, specification proprietary to *brainlife.io* that enables App developers and resource managers to establish programming interfaces, to facilitate the integration of applications with the job scheduling systems (PBS, CONDOR, SLURM, etc) associated with a resource. The interfaces encompass the "`start`" entry point, used to initiate a service, the "`status`" interface, invoked to track the progress of service's job status and the "`stop`" interface, invoked to conclude the execution of service.

Amaretti decentralized resource awareness and prioritization. Amaretti is a meta-orchestration system able to run any App or service published on GitHub and conforming with the ABCD specification. Amaretti is "meta" in the sense that it makes use of the underlying batch-scheduler (job-orchestration) mechanism already existing in computing resources. Amaretti has the ability to run services distributedly on multiple computing resources. In the event that a particular service is enabled on multiple resources, Amaretti utilizes a selection mechanism to choose the optimal resource. For example, a data processing workflow can consist of multiple steps, each implemented in a *brainlife.io* App or service. Amaretti allows sending each step in a sequence of processing steps on a different resource. The same step may be sent to different resources every time it is requested. The outputs resulting from each step are then synchronized after execution is completed. If a user has access to multiple resources on which an App or a service can be executed, Amaretti selects a resource using a series of heuristics. At runtime, Amaretti computes the final resource and decides which resource to use for a service by using the following rules:

1. *Resources scoring.* Resource managers enable Apps or services on a resource. The manager can define a default score for the App, the higher the score the more likely that the resource will be selected to execute a service. Find the default score configured for the resource. If not configured, the resource is disqualified from being used (resource managers must give explicit permission to run the App)
2. *Inter-resources data transfer minimization.* For each App data dependency, the score is incremented by 5 if the resource is used to run the Apps that generate the prerequisite data. This increases the likelihood of reusing the same resource where App runs produced data that is already available on the resource. This approach mitigates data transfer.
3. *Exclusive resource ownership criteria.* An additional ten points are given to a resource if the user possesses exclusive ownership of the resource. Users can define resources only assigned to them. In such cases, rather than utilizing a shared resource, it is advantageous to use the private resource.
4. *Preferred resource ownership criteria.* An increment of fifteen points is added to the score when the resource is designated as the preferred resource to use, as stipulated by the user that submitted the App execution request.
5. *Public resource avoidance.* A project can be configured by users to abstain from using public computing resources. Public resources become ineligible for consideration if the App execution request originates from such a project.
6. *Connection failure.* A resources is disqualified if the resource monitor service detects a connection or server failure.

The resource with the highest score is chosen to execute the task, and a report detailing the rationale behind the resource's selection is added to a file within the service working directory

Tasks. Tasks are the atomic unit of computational work executed on various compute resources. Examples of Tasks are, a job for batch systems, or a vanilla process running on a vanilla VM. Amaretti keeps track of tasks by assigning each one of them a unique process ID.

Service. Any ABCD-compliant GitHub repository is a service for Amaretti. Apps are Amaretti services. When users or the platform submit a task Amaretti retrieves the code service from GitHub. For example, if the user requests to run the Task specified by github.com/brainlife/app-life App, Amaretti will retrieve the code from GitHub, create a copy of the App for that task on a chosen resource and also move.

(Workflow) Instance. Amaretti provides DAG workflow capability by establishing dependencies between tasks. Tasks that depend on parent tasks will simply wait for those parent tasks to complete. All Amaretti tasks belong to a workflow instance (or instance for short).

Resource. Resource is a remote computing resource where Amaretti can securely connect and set up the App execution through the ABCD interface. The resource can be a single computer, a head node of a large high-performance computing cluster, or a submit node for high-throughput computing clusters. The code for the brainlife.io platform is available at https://github.com/brainlife/.

The code used to analyze the thousands of datasets processed in this manuscript is openly accessible on GitHub.com. Below we provide a list of the jupyter notebooks for performing the analyses outlined previously (**Supplementary Table 2**). For this, we provide the jupyter notebook name and the GitHub URL for the respective notebook. Within each notebook, we describe the neuroimaging topic the notebook covers, including structural morphometry (i.e. cortical thickness, surface, area, volume), diffusion profilometry, structural connectivity, functional connectivity, functional gradients, MEEG, and optical coherence tomography (OCT). These notebooks were used to summarize data for different measures and many individual analyses and figures outlined previously. The goal of these notebooks is to document enough information for new users to re-use the notebooks for their own analyses on their own datasets. These notebooks are freely available for use by the greater scientific community.

In addition to providing documentation to the code servicing brainlife.io, we openly release the App code for each App used to analyze the thousands of datasets processed in this manuscript. Below we provide a list of the Apps used for performing the analyses outlined previously (**Supplementary Table 3**). For this, we provide the App

name listed on brainlife.io, the digital-object identifier (DOI) automatically assigned to each app, and the GitHub Repository where the code for the App resides. The goal of this is to increase the transparency of the processing steps performed in this investigation, and for researchers to validate and incorporate into their currently existing workflows.

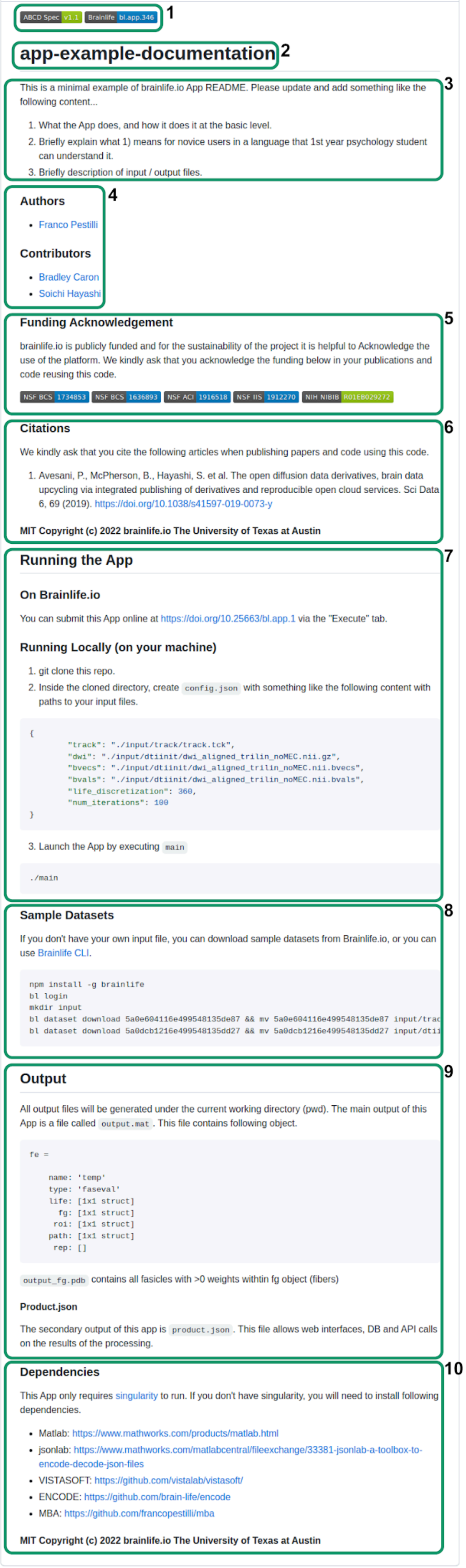

1 ABCD Spec v1.1 Brainlife bl.app.346

2
app-example-documentation

3
This is a minimal example of brainlife.io App README. Please update and add something like the following content...

1. What the App does, and how it does it at the basic level.
2. Briefly explain what 1) means for novice users in a language that 1st year psychology student can understand it.
3. Briefly description of input / output files.

4
Authors

- Franco Pestilli

Contributors

- Bradley Caron
- Soichi Hayashi

5
Funding Acknowledgement

brainlife.io is publicly funded and for the sustainability of the project it is helpful to Acknowledge the use of the platform. We kindly ask that you acknowledge the funding below in your publications and code reusing this code.

NSF BCS 1734853 NSF BCS 1636893 NSF ACI 1916518 NSF IIS 1912270 NIH NIBIB R01EB029272

6
Citations

We kindly ask that you cite the following articles when publishing papers and code using this code.

1. Avesani, P., McPherson, B., Hayashi, S. et al. The open diffusion data derivatives, brain data upcycling via integrated publishing of derivatives and reproducible open cloud services. Sci Data 6, 69 (2019). https://doi.org/10.1038/s41597-019-0073-y

MIT Copyright (c) 2022 brainlife.io The University of Texas at Austin

7
Running the App

On Brainlife.io

You can submit this App online at https://doi.org/10.25663/bl.app.1 via the "Execute" tab.

Running Locally (on your machine)

1. git clone this repo.
2. Inside the cloned directory, create `config.json` with something like the following content with paths to your input files.

```
{
        "track": "./input/track/track.tck",
        "dwi": "./input/dtiinit/dwi_aligned_trilin_noMEC.nii.gz",
        "bvecs": "./input/dtiinit/dwi_aligned_trilin_noMEC.nii.bvecs",
        "bvals": "./input/dtiinit/dwi_aligned_trilin_noMEC.nii.bvals",
        "life_discretization": 360,
        "num_iterations": 100
}
```

3. Launch the App by executing `main`

```
./main
```

8
Sample Datasets

If you don't have your own input file, you can download sample datasets from Brainlife.io, or you can use Brainlife CLI.

```
npm install -g brainlife
bl login
mkdir input
bl dataset download 5a0e604116e499548135de87 && mv 5a0e604116e499548135de87 input/trac
bl dataset download 5a0dcb1216e499548135dd27 && mv 5a0dcb1216e499548135dd27 input/dtii
```

9
Output

All output files will be generated under the current working directory (pwd). The main output of this App is a file called `output.mat`. This file contains following object.

```
fe =

    name: 'temp'
    type: 'faseval'
    life: [1x1 struct]
      fg: [1x1 struct]
     roi: [1x1 struct]
    path: [1x1 struct]
     rep: []
```

`output_fg.pdb` contains all fasicles with >0 weights withtin fg object (fibers)

Product.json

The secondary output of this app is `product.json`. This file allows web interfaces, DB and API calls on the results of the processing.

10
Dependencies

This App only requires singularity to run. If you don't have singularity, you will need to install following dependencies.

- Matlab: https://www.mathworks.com/products/matlab.html
- jsonlab: https://www.mathworks.com/matlabcentral/fileexchange/33381-jsonlab-a-toolbox-to-encode-decode-json-files
- VISTASOFT: https://github.com/vistalab/vistasoft/
- ENCODE: https://github.com/brain-life/encode
- MBA: https://github.com/francopestilli/mba

MIT Copyright (c) 2022 brainlife.io The University of Texas at Austin

Supplementary Figure 1. Brainlife App Github template.

1. App DOI and ABCD specification. **2.** App name. **3.** Description of the App. **4.** Authors and contributors. **5.** Funding Acknowledgement. **6.** Citations. **7.** Instructions for running the app locally, including how to set up the config.json file containing all of the important information for the App including inputs and configuration parameters. **8.** Example datasets that can be downloaded to test the app locally. **9.** The outputs for the App. **10.** The software dependencies subserving the App.

Developing processing Apps for the platform

Here we describe the requirements for developing Apps on the platform. Despite the over 500 apps currently available on the platform, there still exist possibilities for researchers to develop their own processing Apps for performing specific steps that might not already exist on the platform.

The development process for Apps has been streamlined in order to make it as intuitive as possible. Specifically, each App has a set of requirements necessary for the App to be used on the platform. The most important of these requirements involves the creation of a README file outlining all of the important information needed to describe the contents of an App. On Github, we have developed a set of App README templates for App developers to use (Supplementary Figure 1). On the README file, the user must provide information regarding the brainlife.io App DOI and the ABCD specification. In addition, they must also document the app name and a description of what steps the App performs.

Users can also provide information regarding specific authors, coauthors, funding sources, and literature citations in order to provide proper credits for the development of the App. Following these descriptive details, the README should also provide information regarding the usage of the App both on brainlife.io and on local workstations, including descriptions of the inputs, outputs, and software library dependencies of the App. These descriptions found in the README increase the transparency of the App in order to increase the findability and usability of the App.

Supplementary Results 2: Using the platform

Here, we describe the user interface of the platform to help introduce the visual interfaces developed as part of the project. These steps will be described in order of how they would be implemented by a typical researcher designing their own set of experiments using the platform. In addition to visual and text descriptions, we also provide a series of videos documenting each step of the process.

Upon creation of a brainlife.io account, a researcher will first set up a Project within which all of the data processing, storing, and organization will occur (Supplementary Figure 2; Supplementary Table 1). Once their Project is created, users can then update and assign details to the project, including a description of the project, access control to the project, a project README file describing specific information about the project in a machine-readable format, information regarding each participant in the study, and even limit which computing resources the Project will use to process the data.

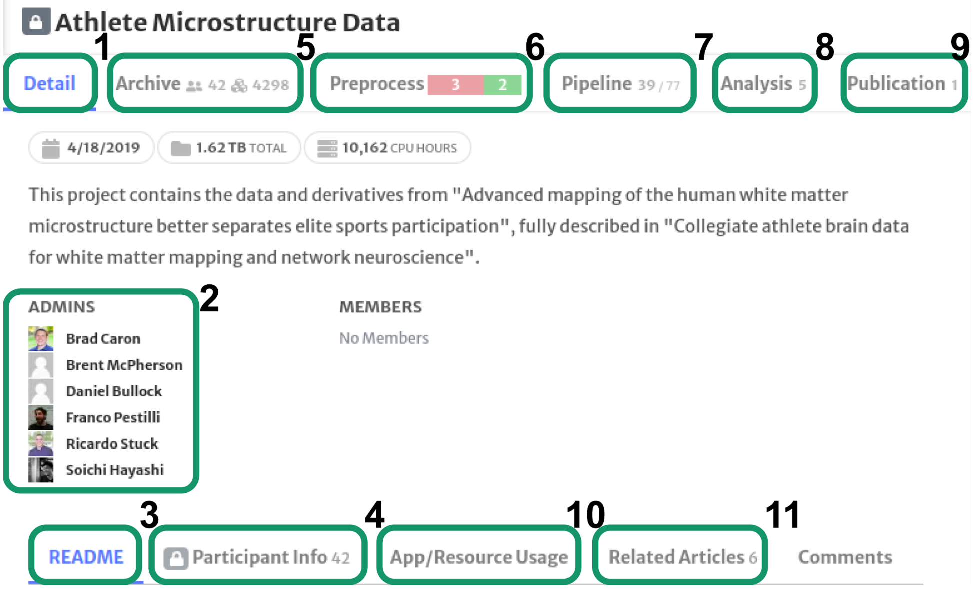

Supplementary Figure 2. Brainlife project landing page.

1. Detail tab containing all of the important details and information describing the Project. 2. Users can add Admins and members for proper project governance. **3.** Projects can have README descriptions, like those on GitHub, to describe important details of the project in a Markdown format. **4.** Participant Info contains tables of demographic information that may be helpful for performing an analysis. This is set and defined by the Administrators of the Project. **5.** Archive tab is where all of the stored files in the form of brainlife datatypes can be found. **6.** The Preprocess tab is where jobs can be launched and monitored. **7.** Pipelines allow the investigator to batch process all of the participants in their project for each App they need to run. **8.** Once statistical features have been extracted, Administrators can access Jupyter Notebooks within the Analysis tab to perform their statistical investigations across all of the participants in the project. **9.** Once the investigators are completed the investigation, they can use the Publication tab to efficiently publish their data and the analysis workflows on brainlife.io. **10.** Whenever a job launches, the App/Resource Usage tab is automatically updated in order to provide provenance tracking of what and where the data processing was performed. **11.** Brainlife.io will search keywords in your project with previously published studies to identify any related articles to your investigation in the Related Articles tab.

Once this information is defined, users are then ready to either import raw datasets they collected or pull datasets that have been openly released. For openly released datasets, users have a variety of options to pull data from including other projects (Supplementary Table 1), or projects hosted on OpenNeuro (Supplementary Table 1). In a similar fashion, users have a variety of options for uploading any newly collected datasets including a built-in GUI (Supplementary Table 1), a CLI (Supplementary Table 1), or through a newly developed sister technology for automated converting of raw scanner data into BIDS-standardized data files known as ezBIDS (Supplementary Table 1). Each of these methods provide a streamlined, efficient way to import data into a new project for future processing and analysis.

Upon importing data into a Project, users can directly interact with the data stored in the Archive tab of the project in multiple ways. First, users can select a data object and visualize the data object using one of the many built-in visualization services for that specific datatype. More importantly, users can then “stage” or move the data from Archive into the Preprocess tab, from which users can select and launch any of the over 400 available Apps (**Supplementary Table 1**). Because Apps on brainlife are “data aware”, users will only be presented with the Apps that take in the staged datatypes that they are designed to work with as inputs ultimately reducing the potential for user error. From the Preprocess tab, users can monitor the status of the App, interact with the data files generated during the App, and visualize the outputs. Once the user is satisfied with the outputs, data objects can be stored back into the Archive tab directly from the Preprocess tab.

This process for running an App is useful under testing circumstances, but may not be appropriate for batch processing of a large number of participants. To facilitate this, users can define Pipeline rules via the Pipeline tab (**Supplementary Table 1**). Within these rules, users specify the inputs including which data objects from the Archive to include or exclude, the configuration parameters required by the App, and the archiving of output objects back into the Archive. Upon launching a Pipeline rule, Amaretti will automatically stage all of the data that matches the input criteria, identify the most appropriate compute resource for running the process, and archive the output data objects back into the project Archive for storage. Outputs from one Pipeline can then be set as inputs to another Pipeline, allowing for the chaining of Apps to develop the overall processing workflow required to get from raw data to the final statistical features of interest needed for statistical analysis.

Once these statistical features of interest are extracted, users can then analyze them directly on the platform via the Jupyter Notebooks provided by brainlife.io (**Supplementary Table 1**). To facilitate this, a certain subset of all datatypes that correspond to statistical features of interest are stored in a secondary warehouse, which can be directly loaded via the Jupyter Notebooks. This ultimately reduces the number of potential data objects and storage size of the objects required by brainlife.io to move into the Notebooks, ultimately making the process more efficient for users. Common subsets of functions, including those useful for loading data into the Notebooks, have been packaged into a Python package *pybrainlife* that can be imported directly into the Notebooks and used to load and compile an entire study’s worth of statistical features. Upon completion of the analyses, these Notebooks can be directly published and/or pushed to GitHub in order to increase the scientific transparency of the project. In addition to the publication of the Notebooks, brainlife.io automatically keeps track of each individual step performed to obtain a specific datatype (i.e. provenance; **Supplementary Table 1**). This visualizer contains all of the information a user might need to validate that the proper steps were taken, and for any outsider users or reviewers to rerun their analysis steps for purposes of replication. Finally, upon completion of processing and analysis, researchers can Publish their datasets, Pipeline rules, and Analysis notebooks directly on the platform via the Publications tab (**Supplementary Table 1**). Finally, a single record containing data objects, Apps, and Jupyter Notebooks used in a study can be made publicly available outside the platform in a single record addressed by Digital Objects Identifiers (DOI) [58]. Whereas all other existing systems provide users with technology to track analysis steps manually or require the use of coding, *brainlife.io* tracks automatically and does not require coding. This automation technology lowers the barriers of entry to reproducible and transparent large-scale neuroimaging data analysis.

End-to-end reproducible scientific workflow

Neuroimaging investigations involve a common workflow from data collection to study publication (**Figure 1e**). Data are first either collected from neuroimaging measurement systems, including MRI and MEG scanners. Following collection, data is then converted to standardized file formats before they can be used by the researcher. From here, common artifacts are removed from the data in a series of preprocessing steps. Once the data is cleaned, models can be fit, brain structures can be segmented, and quality assurance assessments are performed. If any mistakes occurred in the previous steps, adjustments can be made to each individual step in order to increase data quality. Only once the data are of high enough quality are statistical brain features of interest extracted, and statistical analyses are performed on the extracted features. Final results, data, and code are then published to the greater scientific community to increase transparency and data gravity of the investigation. *brainlife.io* serves each step following data collection, with each step of the workflow tracked in order to increase reproducibility (**Figure 1f**).

brainlife.io automatically tracks all actions performed by researchers during data analysis. Data object IDs, Apps versions, and parameter sets used to launch an App, resources used, error logs, etc. are all tracked automatically by brainlife.io. The full sequence of steps from data import to preprocessing, analysis, and publication is captured by the platform and is used to build a record of all the actions researchers performed while implementing a data analysis study. A graph describing provenance metadata for each Datatype can be visualized using the provenance visualizer and downloaded (see **Figure 1f** and **Supplementary Table 1**). The graph can be downloaded as a JSON file from the GUI window that describes the properties of a data object in a project.

In addition to the data provenance graph, brainlife.io also generates automatically a script that allows users to reproduce the workflow used to create a specific data object: `reproduce.sh` (**Supplementary Table 1**). The script can be downloaded from the GUI window that describes the properties of a data object in a project, and it encodes the series of steps used to generate the data object. The script can be used by installing the CLI (e.g., on a cluster, server, or local computer) and has only a few system requirements, such as `git`, `docker`, and `singularity`. In addition to this file, a `boutiques`[59] descriptor file is automatically generated to reproduce a brainlife.io workflow on different systems. This is an experimental component of brainlife.io, `boutiques` descriptors in principle allow interoperability between brainlife.io, CBRAIN, VIP[60], and Pegasus[61]. We plan to further develop this feature in the future.

In sum, brainlife.io is meant to help students, researchers, and clinicians to perform the end-to-end reproducible neuroimaging workflow by providing the user the following services for free: data archiving and storage, file formatting & standardization, provenance tracking, access to & management of HPC environments, job submission on HPC environments, coding/development/testing of processing code for data processing, containerization of software libraries, data processing, statistical feature extraction, compilation of statistical features in tidy data formats, data & code publication, quality assurance, mechanisms for collaboration, and education.

Supplementary Results 3: Platform utilization

brainlife.io was developed with a FAIR model and made available worldwide. Any researcher can create an account on *brainlife.io*, although all new accounts are reviewed by the project team. *brainlife.io* first became publicly available in 2018. We tracked the usage of *brainlife.io* in the past 80 months. The platform community, utilization, and research assets have grown steadily since project inception (**Extended Data Figure 2**). At the time of writing, over 2,341 users across 43 countries have created a *brainlife.io* account. Over 1,266 active users submitted more than 100 jobs per month (**Extended Data Figure 2a**). There were 3,439 data management Projects. The *brainlife.io* developers' community had implemented 530 data processing Apps comprising 131,235,554 lines of code (top 50 apps), and these had been used to process over 270 TBs of data for a total of 3,951,372,037,289 hours of compute time. Apps success rate on average has been 65.4% across 6,710,091 total job submissions (the estimates contain high-failure rate App test-calls). This level of interest and reach, even prior to a formal publication describing the platform, is a testament to *brainlife.io*'s potential for growth and impact.

Researchers ranging from undergraduate students to faculty have already used *brainlife.io* (**Extended Data Figure 2b**). The Apps spanned various aspects of the neuroimaging data lifecycle. The most frequently used Apps pertained to tractography (22%), model fitting (15%), and ROI generation (12%). Community-developed software libraries provided the foundations for data processing Apps, including Nibabel, Freesurfer, FSL, DIPY, MRTrix, Connectome Workbench, and MNE-Python. Terabytes of data have been uploaded (72%) or imported from OpenNeuro.org (22%), the Nathan-Kline Institute data sharing projects (3%; [51,53,62]), and other sources. Early community attention and adoption preceded this publication describing the project and platform. The worldwide platform access highlights the global need for technology like *brainlife.io* (**Extended Data Figure 2c**).

In sum, *brainlife.io* and its user community are highly engaged in providing innovative training and education opportunities for the next generation of students, postdocs, and clinicians interested in the intersection between neuroscience, data science, and information. The platform allows new students and educators to access many complex data files and analysis methods with minimal overhead. Educators have started using *brainlife.io* to teach neuroscience and data science concepts in the classroom, and courses have been organized in Europe, the USA, Canada, and Africa. These courses introduce basic concepts and teach students how to perform neuroimaging investigations without the requirement of programming or computing expertise. The skills that can be learned using the platform include data preprocessing, quality assurance, and statistical analyses. Integrative data

management and analysis provide opportunities for educators and students in under-resourced institutions or countries to perform research and teach neuroscience with hands-on experience.

Apps performance evaluation

Brainlife.io, like any technology, is not *failure-proof*. To examine the rate at which brainlife.io Apps fail, we collected data regarding the failure rates of all Apps across the platform. Since the beginning of the platform, jobs processed on brainlife.io have had a 34.6% failure rate across 6,710,091 submissions, with half estimated to be due to initial App testing and development (**Supplementary Figure 3**).

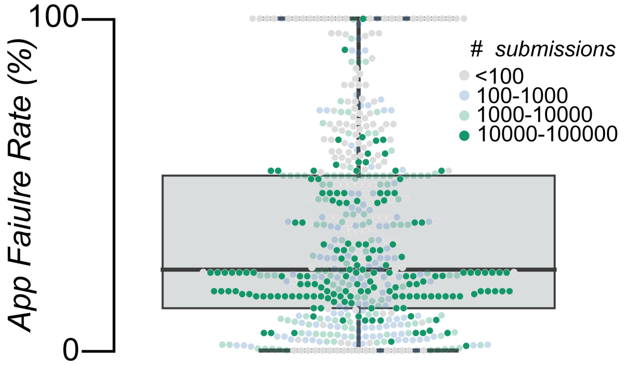

Supplementary Figure 3. Brainlife.io processing is not error-proof.

Distribution of brainlife.io App failure rates (percentage) across all 568 Apps and their respective submissions. Box-and-whisker plot indicates the overall average failure rate across all Apps (*dark black line*), 25th and 75th percentiles (*box*), and overall range (*whiskers*). Each dot is an individual App's failure rate. Colors represent the number of submissions for each App (*gray*: 0-100 submissions, *light blue*: 100-1,000 submissions, *light green*: 1,000-10,000 submissions, *dark green*: 10,000-100,000 submissions).

Supplementary Results 4.1: Platform testing

The effectiveness of the technology to provide good quality results were evaluated. We performed system load experiments by processing large amounts of data and evaluating the results obtained. These experiments were performed to demonstrate the ability of the platform to serve accurate data processing and analysis at scale.

Experiments were performed to demonstrate the ability of the platform to provide accurate data processing and analysis at scale. The experiments focused on the four axes of scientific transparency: data processing external validity (DPEV), reliability, reproducibility, and replicability.[63,64] Four data modalities (sMRI, fMRI, dMRI, MEG) were evaluated using, among others, the test-retest $HCP_{TR,}$ [65] the Cam-CAN,[66] the HBN,[62] and the ABCD[67] datasets (See **Supplementary Table 6**). In total, data from over 3,200 participants across 12 datasets were processed. Extracted brain features included cortical parcel volumes, white matter tract profilometry, functional and structural network properties, functional gradients, and peak alpha frequency. Over 193,000 data objects were generated for the experiments in 147,988 hours, utilizing 22 Terabytes (TB) of storage (**Supplementary Figure 4**).

All the data processing was completed in less than 4 months using brainlife.io. We estimated that using a more traditional in-lab personal workstation setup, it would have taken at least 36 months just to preprocess the same data, roughly a 9x reduction in computational time using *brainlife.io*. We note that these are conservative estimates under the assumption that an average of 6-8 sequential 1-hour steps (or Apps) were performed by brainlife.io for each data modality. This assumption is an estimate and not exact given that the platform can run Apps in parallel unless Apps are waiting for dependencies. Finally, given the current average hard-drive storage per single workstation (2TB), we estimated that roughly 11 workstations or hard drives would be needed to store all the data. The *brainlife.io* Apps used for the experiments are reported in **Supplementary Table 3**. Post-processing analyses were performed using *brainlife.io*-hosted Jupyter Notebooks (see **Supplementary Table 2**).

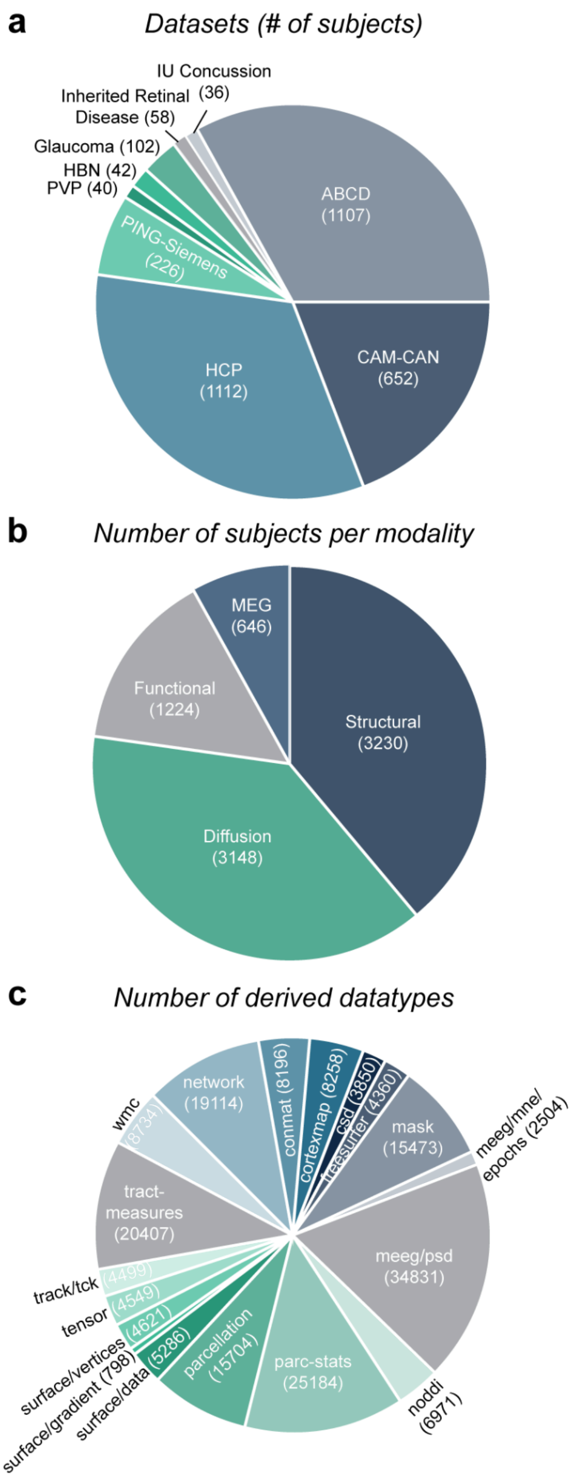

Supplementary Figure 4. Overview of data used for the study.

a. Number of subjects across all datasets examined. **b** The number of subjects per imaging modality. **c.** The number of brainlife.io datatypes (i.e. *freesurfer, parc-stats, tractmeasures, track/tck, NODDI, tensor, csd, mask, network, conmat, parcellation, cortex map, wmc, meeg/psd, meeg/mne/epochs, surface/data, surface/vertices, surface/gradient*) derived across all subjects and datasets examined. See https://brainlife.io/datatypes for a description of the data types

Data processing external validity (DPEV) was defined as the ability of data processed on *brainlife.io* to accurately reflect brain properties proficiently processed by other teams. DPEV was estimated for four data modalities (sMRI, dMRI, fMRI, and MEG) and five brain features (brain areas volumes, major white matter tracts fractional anisotropy, resting state functional connectivity, resting-state function gradients, and MEG peak alpha frequency). Features values obtained using *brainlife.io* Apps were compared against data preprocessed by data originators, specifically the HCP consortium or Cam-CAN project team (**Extended Data Figure 3**). For the structural, diffusion, and functional MRI modalities, statistical features extracted from data preprocessed using *brainlife.io*

Apps were compared directly to features extracted from data provided by the HCP Consortium. For the MEG modality, statistical features extracted from data preprocessed using *brainlife.io* Apps were compared directly to features extracted from data preprocessed by the Cam-CAN consortium. Cortical area volume estimates on 148 parcels were obtained using *brainlife.io* Apps A0, A462, A23, A272, and A464 and compared to corresponding estimates provided by the HCP consortium (**Extended Data Figure 3**; $r_{validity}$=0.98, $rmse_{validity}$=570.54mm^3). See **Extended Data Figure 4** for additional parcellations (hcp-mmp-b) and measures (i.e. cortical thickness, and surface area). Fractional anisotropy (FA) in 61 white matter tracts was estimated using *brainlife.io* Apps A68, A238, A297, A305, A188, A195, and A361 using the raw and minimally preprocessed HCP_{TR} dMRI data (**Extended Data Figure 3**; $r_{validity}$=0.95, $rmse_{validity}$=0.018). These Apps were used to process either type of data, with the exception of A68,[5] for which only raw data was used. See **Extended Data Figure 5** for additional measures (i.e. axial diffusivity (AD), radial diffusivity (RD), and mean diffusivity (MD)). Functional connectivity estimates between 117^2 nodes-pairs [68] were compared between raw and minimally preprocessed HCP_{TR} fMRI data using *brainlife.io* Apps A604 and A574 (**Extended Data Figure 3**; $r_{validity}$=0.89, $rmse_{validity}$=0.12). In addition, functional gradients [69,70] were computed on 400 nodes estimated on raw and minimally processed HCP_{TR} fMRI data (**Extended Data Figure 3**; $r_{validity}$=0.59, $rmse_{validity}$=0.036). Finally, the peak alpha frequency values were compared between Cam-CAN and *brainlife.io* processed MEG data using *brainlife.io* Apps A476 and A531 [71,72] (**Extended Data Figure 3**; $r_{validity}$=0.94, $rmse_{validity}$=0.30 Hz). Overall, the results show strong similarity in feature estimates between data processed on *brainlife.io* versus those processed by external groups (functional gradients demonstrated the lowest validity and data processing-type dependency based on fMRI preprocessing procedures [73]).

Data processing reliability (DPR) was defined as the ability to produce highly similar results on *test* and *retest measurements* within a study participant. DPR was estimated for the four data modalities and five brain features used above to estimate DPEV. Brain features estimated using *brainlife.io* Apps on *test* and *retest measurements* (HCP_{TR} dataset) or median splits data (Cam-CAN MEG) were compared. Specifically, for the structural, diffusion, and functional MRI modalities, statistical features extracted from HCP Test data were compared directly to features extracted from HCP Retest data. For the MEG modality, a median split of the timeseries was performed and statistics from each split were compared against each other. Reliability estimates of brain area volumes, major tracts FA, networks FC, functional gradients, and Peak Alpha Frequency were obtained (see **Extended Data Figure 3**). DPR varied between $r_{reliability}$=0.99 and 0.73, with sMRI and dMRI demonstrating the highest reliability ($r_{reliability}$=0.99, 0.93, respectively). See also **Supplementary Table 4** for a full report of all correlation values obtained in all brain features. The results show strong reliability of most of all the pipelines with the fMRI reliability being lowest, this is consistent with previous reports [74].

Specifically, cortical parcel volumes from the test and retest dataset of HCP_{TR} were obtained using A23, A272, and A464 brainlife.io Apps (see **Supplementary Table 3**) and compared (**Extended Data Figure 3**; $r_{reliability}$=0.99, $rmse_{reliability}$=278.11mm^3). See **Extended Data Figure 4** for additional parcellations (hcp-mmp) and measures (cortical thickness, surface area, volume). Mean FA from 61 white matter tracts was estimated independently for *test* and *retest* HCP_{TR} dMRI data using A238, A297, A305, A188, A195, and A361. The average FA for each tract was compared between test and retest conditions ($r_{reliability}$=0.93, $rmse_{reliability}$=0.017) (**Extended Data Figure 5**). Functional connectivity estimates between 117^2 nodes-pairs were estimated using the test and retest HCP_{TR} fMRI data using A23, A369, and A532 (**Extended Data Figure 3**; $r_{reliability}$=0.73, $rmse_{reliability}$=0.19). In addition, functional gradients were computed on 400 nodes estimated on test and retest HCP_{TR} fMRI data using A604 and A574. The average primary gradient within each node was compared between datasets (**Extended Data Figure 3**; $r_{reliability}$=0.85, $rmse_{reliability}$=0.026). Finally, the frequency of the amplitude peak (between 8 and 13 Hz from the occipital magnetometers and gradiometers) was estimated from two median splits of Maxwell-filtered Cam-CAN MEG data using A529 and A531. Peak alpha frequency values were compared between the two datasets ($r_{reliability}$=0.85, $rmse_{reliability}$=0.48 Hz; **Extended Data Figure 3**). All estimated validity and reliability estimates are reported in **Supplementary Table 4**.

We also performed computational reproducibility (CR) experiments (see **Extended Data Figure 5**). These experiments demonstrated the similarity in estimates produced by *brainlife.io* Apps when used twice to process the same dataset. Given the use of containerization technology for the Apps, this test was expected to return high correlation values. Indeed, all correlations were above 0.99, demonstrating high consistency. These experiments demonstrate the ability of the platform to conduct valid, reliable, and reproducible data processing and analysis at scale across multiple data modalities and brain features.

Specifically, cortical parcel volumes were estimated twice from the minimally processed HCP_{TR} data (N_{sub} = 44) using A272. Volume estimates between the repeat run were compared (**Extended Data Figure 5;** $r_{reproducibility}$ = 0.99, $rmse_{reproducibility}$ = 34.22 mm^3). Mean FA in 61 white matter tracts was estimated from the minimally processed HCP_{TR} data (N_{sub} = 43) using A361. The average FA for each tract was compared between repeated runs (**Extended Data Figure 5**; $r_{reprodubility}$ = 0.99, $rmse_{reprodubility}$ = 0.011). Functional connectivity estimates between 117^2 node pairs were estimated using the minimally processed test HCP_{TR} data (N_{sub} = 32) using A532. Average node connectivity was compared between repeated runs (**Extended Data Figure 5;** $r_{reproducibility}$ = 1.0, $rmse_{reproducibility}$ = 0.0). In addition, functional gradients were computed on 400 nodes estimated from the Cam-CAN data (N_{sub} = 613) using A574. Finally, primary gradient values were compared between repeated runs (**Extended Data Figure 5** $r_{reproducibility}$ = 0.99, $rmse_{reproducibilty}$ = 0.03). Finally, the peak alpha frequency (Hz) was estimated from the Maxwell-filtered MEEG Cam-CAN data (N_{sub} = 501) using A531. Peak alpha values were compared between repeated runs (**Extended Data Figure 5**; $r_{reproducibility}$ = 0.99, $rmse_{reproducibility}$ = 0.0002).

Quality control at scale

A critical aspect to democratizing big data neuroscience is the ability of investigators to perform quality assurance (QA), because there is no value in increasing dataset size unless quality can be assured for each dataset. State-of-the-art approaches provide users with the ability to assess quality after data processing is compiled into QA reports [31,32,75–78], or through the use of citizen science [79]. The brainlife.io platform supports visualization of the QA reports outputted by state-of-the-art processing pipelines (A160, A246, A160, A462, A423, A399), as well as via QA images, which can be assessed by individuals or groups. Here we propose an additional approach to QA via *normalized reference ranges*, in which brain properties derived from many participants, modalities, and sources of variability are collated together for quick identification of abnormal brain derivatives [80].

Reference ranges are often used in vision science to provide a reference for a measurement, [80] and a similar approach was integrated within the *brainlife.io* data processing interface. To test it, the mean, first, and second SD were estimated (via multiple Apps) for four brain features (tractmeasures, parc-stats, networks, PSD) using the HCP_{s1200}, Cam-CAN, and PING datasets. Volume measures were estimated using A464, A462, A272, and A379. For diffusion MRI data, the average FA for each of the white matter tracts segmented for each participant was compared to the participant age at scan acquisition on a per-structure basis. Tract average FA values were estimated using A361. In addition to white matter tract FA, average FA within cortical regions was computed using A383. For resting-state functional MRI connectivity, the average within-network connectivity values, defined as the average connectivity values between all of the nodes within each resting state network of the Yeo17 parcellation, was compared to the participant's age at scan acquisition. Network connectivity matrices were estimated using A532. For resting-state functional gradients, the cosine distance of the primary gradient for each of the resting state networks in the Schaffer parcellation was compared to the participant's age at scan acquisition. Gradients were mapped using A574. Finally, for MEG data, the peak frequency in the alpha band across all nodes was compared to the participant's age at the time of acquisition. Peak frequency was estimated using A531. For structural and diffusion MRI data, data from all three data sources (HCP_{s1200}, Cam-CAN, PING) was used. For the functional MRI data, data from only the HCP_{s1200} and Cam-CAN data sources were used. For the MEG data, only the data from the Cam-CAN data source was used.

For each of the four brain features, the estimated mean and estimated s.d. (referred to here as *Reference ranges*) are automatically calculated on the *brainlife.io* platform. That is, when a researcher uses an App to estimate one of the four features, the values of the researcher's dataset are automatically overlaid on top of the mean, first, and second s.d. marks provided as a reference by *brainlife.io.* In this way, the mean and variability can be used by researchers to efficiently judge whether a recently processed dataset returned appropriate values. For example, reference datasets can be used to detect outlier data (**Extended Data Figure 6**). Example reference datasets for four Datatypes are in **Extended Data Figure 6** and an example of platform interfaces reporting these reference datasets is shown in **Supplementary Figure 5**. These reference ranges are an additional source for quality assurance, alongside other options for QA such as online data visualization, the automated generation of images and plots from the processed data as well as the detailed technical reports from major BIDS Apps such as fMRIprep, QSIPrep, MRIQC, Freesurfer [31,32,78,81].

To generate the reference ranges, the brain properties derived from the three datasets (PING, HCP_{s1200}, and Cam-CAN) and four data modalities in 1,751 participants generated for the load testing of the platform (as

described in the previous sections) were curated (removed of outliers) and collated for *brainlife.io* datatype. For each datatype, a single JSON file was created reporting the mean and ±1 and 2 standard deviations of the outlier-removed measure (e.g., the volume of a brain parcel, fractional anisotropy of a white matter tract, functional connectivity of a network, power-spectrum density across MEG sensors). The JSON files were saved on a repository (github.com/brainlife/reference) and the brainlife.io datatype validator service made use of the JSON to automatically visualize a plot of the data. We call these JSON files reference datasets. Users utilizing Apps (A272, A463, A483, A361, A530, A531, A532) that generate datatypes for which a reference dataset was created will find the values of the features estimated by the App on any new dataset overlaid on top of the corresponding reference dataset (see **Supplementary Figure 5**).

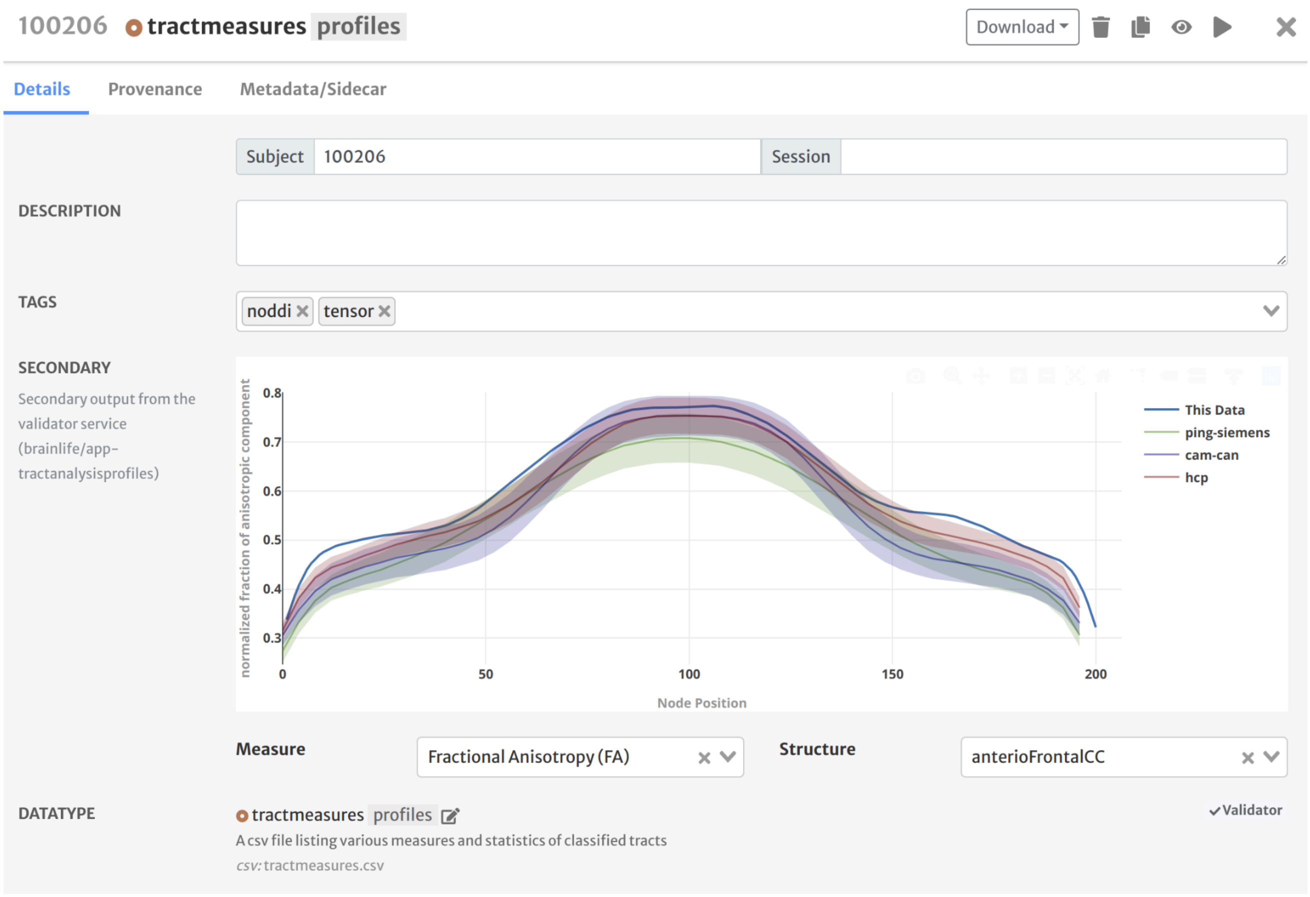

Supplementary Figure 5. brainlife.io interface can visualize reference datasets.

Validation services for datatypes containing statistical feature information automatically generate a visualization of newly generated data (*blue line*) overlaid on reference dataset ranges for the three data sources used to generate reference datasets (i.e. HCP_{S1200} (*red*), PING (*green*), CAN (*purple*). These reference ranges can be used to quickly assess the quality of the estimated statistical features of interest. Data are presented as mean values +/- SEM.

Supplementary Results 4.2: Platform utility

Evaluation of the scientific utility of the platform was performed on over 2,000 participants across three large datasets with participant ages spanning over 7 decades—PING (Pediatric Imaging, Neurocognition, Genetics), HCP_{s1200}, (Human Connectome Project Young Adult 1,200) and Cam-CAN (Cambridge Center for Ageing Neuroscience). Multiple brain features were derived, including fractional anisotropy of cortical parcels and within-network functional connectivity of individual Yeo17 networks. Specifically, for structural MRI data, the volumes of the cortical and subcortical structures segmented for each participant were compared to their age at the time of scan acquisition on a per-structure basis. Volume measures were estimated using A464, A462, A272, and A379. For diffusion MRI data, the average FA for each of the white matter tracts segmented for each participant was compared to the participant age at scan acquisition on a per-structure basis. Tract average FA

values were estimated using A361. In addition to white matter tract FA, average FA within cortical regions was computed using A383. For resting-state functional MRI connectivity, the average within-network connectivity values, defined as the average connectivity values between all of the nodes within each resting state network of the Yeo17 parcellation, was compared to the participant's age at scan acquisition. Network connectivity matrices were estimated using A532. For resting-state functional gradients, the cosine distance of the primary gradient for each of the resting state networks in the Schaffer parcellation was compared to the participant's age at scan acquisition. Gradients were mapped using A574. Finally, for MEG data, the peak frequency in the alpha band across all nodes was compared to the participant's age at the time of acquisition. Peak frequency was estimated using A531. For structural and diffusion MRI data, data from all three data sources (HCP_{s1200}, Cam-CAN, PING) was used. For the functional MRI data, data from only the HCP_{s1200} and Cam-CAN data sources were used. For the MEG data, only the data from the Cam-CAN data source was used.

To assess the relationship between each of the measures and age within each structure investigated (**Fig 2a; Extended Data Figure 6)**, a quadratic model ($y_{feature} = ax_{age}^2 + bx_{age} + c$) was fit across all of the data, and a linear regression was fit within each data source, using functions from scikit-learn[82] (ages 3 to 88): $y_{feature} = ax_{age}^2 + bx_{age} + c$, ($R^2$=0.152±0.0773 s.d.) (**Fig 2a; Extended Data Figure 6).** Mean quadratic term (*a*) across all data modalities was negative (-0.0514 ± 0.111 s.d.). Two additional examples are presented in **Extended Data Figure 6j**, specifically the average fractional anisotropy (FA) of cortical V1 (**Extended Data Figure 6**) and the within-network average functional connectivity within the default mode (A) network derived from the Yeo17 atlas (**Extended Data Figure 6**). The quadratic model (R^2=0.12 ± 0.015 s.d.) for these two examples demonstrated the expected inverted U-shape trajectory, with the mean quadratic term (*a*) across each data modality being negative ($-3.70\times10^{-6} \pm 6.60\times10^{-6}$ s.d.).

Supplementary Results 4.3: Replication and generalization

In addition to the replication experiments, five sets of generalization experiments were performed (**Fig 2b; Extended Data Figure 7**). First, we tested *brainlife.io*'s ability to replicate scientific results from five previous studies [83–85]. A key finding from each previous study was identified as the target found to be reproduced. We then followed the processing methods as outlined in the original study but performed these processing methods using *brainlife.io* Apps. Post-processing analyses were performed in line with the original study using *brainlife.io*-hosted Jupyter Notebooks (see **Supplementary Table 2**). Replicability success was measured by comparing trends in the data obtained with brainlife.io Apps and those reported in the original study.

Replicability was defined as the ability to reproduce individual experiments already published by other members of the scientific community. Within replicability are two pillars: the ability to reproduce results within the *same* dataset, and the ability to generalize results to *new* datasets. Three sets of experiments were performed to assess the ability of the platform to replicate previously published findings. The first experiment attempted to replicate a reported negative correlation between a cortical region's thickness and its tissue orientation organization within the HCP_{s1200} dataset[83]. Cortical regions found within the HCP multi-modal parcellation (hcp-mmp) parcellation were first mapped to each participant's Freesurfer surfaces using A23. Brainlife apps A464, A462, A272, and A379 were then used to map and estimate each region's cortical thickness and orientation dispersion index (ODI), respectively. The relationship between ODI and cortical thickness was assessed by computing the correlation between these values across all parcels within the hcp-mmp parcellation (**Fig 2b; Extended Data Figure 8**). A negative trend was identified replicating results in [83] ($r_{HCP\text{-}brainlife}$ = -0.43 vs. $r_{original}$).

The second experiment attempted to replicate the improved ability to segment the Inferior Longitudinal Fasciculus from the HCP_{s1200} dataset (**Extended Data Figure 8**) [40]. The Right Inferior Longitudinal Fasciculus (ILF) was segmented from the HCP_{s1200} dataset using an automated segmentation algorithm (A174). The same improved ability of tract segmentation was obtained (AUC_{LAP} = 0.77, $AUC_{NN\_DR\_MAM}$ = 0.66). The third study used to assess replicability investigated the performance of an automated hippocampal subfield segmentation as compared to hand-drawn regions of interest (ROIs)[86]. The original implementation was performed with a dice coefficient ranging from 0.525-0.823. An App (A262) was created to implement this segmentation on brainlife. The method was implemented on participants from the UPENN-PMC dataset. Improved model performance was obtained for segmenting hippocampal subfields (**Extended Data Figure 8**; dice range = 0.838-0.945).

In addition to the replication experiments, three sets of generalization experiments were performed. The first experiment attempted to generalize the same relationship between a cortical region's thickness and orientation dispersion index found within the HCP_{s1200} dataset to the Cam-CAN dataset (**Fig. 2b; Extended Data Figure 8**). *brainlife.io* Apps A464, A462, A272, and A379 were then used to map and estimate each region's cortical thickness and orientation dispersion index (*ODI*), respectively. The relationship between ODI and cortical thickness was assessed by computing the correlation between these values across all parcels within the hcp-mmp parcellation. A negative trend of about half the magnitude of the original was estimated ($r_{Cam\text{-}CAN\text{-}brainlife}$ = -0.28 vs. $r_{original}$).

The second and third experiments attempted to generalize a relationship between the average quantitative anisotropy (QA) and fractional anisotropy (FA) of the left and right uncinate with the presence of stressful life events as an adolescent (**Fig. 2c; Extended Data Figure 8**). The second experiment assessed tract organization within the UF of 42 participants from within the HBN dataset using A423 to extract the UFs and to map QA to each, respectively. These values were then compared to the number of negative life events as reported on the Negative Life Events Schedule (NLES) collected by the HBN group. A negative relationship as identified using a linear regression model between UF QA and number of stressful life events was identified (**Fig. 2c; Extended Data Figure 8** r_{HBN_LEFT} = -0.38, p-value = 0.018; r_{HBN_RIGHT} = -0.39, p-value = 0.0156). The third experiment attempted to find the same relationship using FA within 1,107 participants from the ABCD dataset. For this, an end-to-end white matter processing pipeline composed of A68, A238, A297, A305, A188, A195, and A361 was used to extract the UF and to map FA to each tract. These values were then compared to the measure of early life stress was estimated as a composite score by z-scoring separately and then summing across the following questionnaires: traumatic life events reported by the parent, environmental and neighborhood safety reported by both parent and adolescent, and the Family Environment Scale-Family Conflict Subscale Modified from PhenX reported by both parent and adolescent [87]. A negative relationship as identified using a linear regression model between UF FA and the composite score was estimated in the left- and right-UF (**Fig. 2c; Extended Data Figure 8** r_{ABCD_LEFT} = -0.06, p-value < $9.4x10^{-5}$; r_{ABCD_RIGHT} = -0.04, p < 0.004).

Supplementary Results 4.5: Detecting disease

The two tests evaluated the platform's ability to identify human disease biomarkers. eye disease (Choroideremia and Stargardt's disease), and matched controls were used (**Fig. 2d**). It is important to note that *brainlife.io* only provides tools for performing analyses to potentially identify clinical biomarkers, however it does not provide diagnostic tools nor do the performed analyses qualify as a diagnostic procedure.

Changes in the white matter of the optic radiation (OR) as a result of eye disease have been reported.[46,88–91] We set out to test the ability of *brainlife.io* Apps to detect similar changes in the OR white matter tissue in two eye diseases for which OR white matter changes have not previously been reported. Individuals with Stargardt's disease (a deterioration of the retina initiating in the central fovea), and Choroideremia (retinal deterioration initiating in the visual periphery), were compared to healthy controls. Retina photoreceptor complex thickness was estimated in the fovea and peripheral using optical coherence tomography (0-1 and 7-90 degrees of visual eccentricity, respectively; **Fig 2d**) using *brainlife.io* App A346.

Choroideremia patients showed photoreceptor complex thickness comparable to healthy controls in the fovea, but deviated in the periphery (**Fig 2d**). The trend was opposite for Stargardt's patients. *brainlife.io* Apps were developed to automatically separate OR bundles projecting to different visual eccentricity in cortical area V1. Average FA profiles for each patient group and controls were estimated for OR fibers projecting to the fovea or periphery using a series of *brainlife.io* Apps (A273, A462, A187, A414, A233, A361, A68, A238, and A346).[92] [93,94] Results show a reduction in FA in the component of the OR projecting to the fovea (but not the periphery) in Stargardt's patients (**Fig 2d**, blue), and the opposite pattern (OR fibers projecting to the periphery had lower FA than controls) in Choroideremia patients (**Fig 2d**, blue). These results demonstrate the ability of the platform technology to detect disease biomarkers.

Supplementary Results 5: Public services for promoting transparency and data gravity in neuroscience research.

In the previous section, we described the system architecture for the platform and evaluated the platform through a number of experiments. These components and architectures were implemented in order to reduce barriers of entry to performing neuroimaging investigations and to ultimately increase data gravity and representation in neuroscience.

The platform was developed with public funding to promote brain science research and education. The project leadership and advisory team recognize the importance of ensuring proficient data governance is considered an integral part of data processing. Data governance is defined as the principles, procedures, technologies, and policies that ensure acceptable and responsible processing of data at each stage of the data life cycle.[9] It comprises the management of the availability, usability, integrity, quality, and security of data.[9]

The landscape of neuroscience research is changing, often crossing international borders[9]. As a result, compliance with local and international mandates for data privacy and sharing will ultimately require moving data management and processing to secure and professionally managed systems. The platform interface provides mechanisms to support some aspects of this international data management process. For example, it provides Data Use Agreements (DUA) templates and text fields for the data managers to add additional provisions aligned with their specific data protection requirements and for sharing data in their relevant jurisdiction from where the data originate. Yet, brainlife.io does not claim to address the requirements of all applicable regulations, such as the European Union's General Data Protection Regulation (GDPR) and the Health Insurance Portability and Accountability Act (HIPAA) in the United States or the Personal Information Protection Law (PIPL) in China, however, the use of a DUA is a critical organizational mechanism to protect the personal information of research participants. The ultimate responsibility of ensuring the information security (confidentiality, integrity, availability) and the data protection and privacy rights of the research participants rests on the data controllers (the project owners, project managers/or data providers in a research project). The data controllers must ensure that the study project conforms with the legal rules and regulations applicable to their project and the collection, processing, transfer and sharing of research data in their jurisdiction and the jurisdiction of the individuals whose data are being used for the study project. brainlife.io provides a secure platform for the datasets and is liable in cases of security breaches caused by *brainlife.io's* negligence, omissions, or intentional misconduct in terms of security measures. The data providers are responsible for any breach of data protection, privacy, and confidentiality requirements applicable to research data as described in the Acceptable Use Policy (AUP)[95]. This may happen in case inadequate pseudonymization methods are used when a user uploads research data on brainlife.io. The platform is registered on fairsharing.org, datacite.org, and nitric.org, it is recommended by the International Neuroinformatics Coordinating Facility (incf.org/infrastructure/brainlife), and it can serve the U.S. National Institutes of Health in the United States data deposition and sharing mandate[96].

Data gravity is the ability of datasets to attract utilization. Neuroimaging research within the larger neuroscience arena has led the way for increasing data gravity. A long and growing list of tools orchestrated under a general label of open science have been, and are being, developed to support and facilitate data utilization and access. These tools can be divided into four main categories: software library, data archives/database systems, data standards, and computing platforms (see **Platform architecture** and **Supplementary Table 1**). The unique ability of *brainlife.io* to use data from multiple modalities (MRI, MEG, EEG) is an important feature that increases data gravity by connecting traditionally siloed neuroimaging research sectors. Future opportunities for expanding data types managed by the platform are possible, given existing mechanisms for adding Datatypes. Finally, improving connection with major archives and platforms such as OpenNeuro.org, DANDI, NeuroScout, NeuroDesk, and neurosynth.org, would aid in implementing the vision of a global interoperable ecosystem for FAIR, accessible, and democratized neuroscience.

The goals outlined for *brainlife.io* coincide with a push within the neuroimaging community to increase data gravity and representation by providing standardization of data formatting, software libraries, and computing resources. From this push has come an ever-growing list of publicly available services and platforms for increasing data gravity in neuroimaging. However, there currently exists only one compiled list of the services available [97]. To address this, and to help increase transparency in neuroscientific research, we provide a

non-comprehensive list of currently available services and platforms for increasing data gravity across the greater neuroimaging community (**Supplementary Table 5**). This list is not designed to cover all currently available services and platforms, but to provide a sense of the scope of available technologies developed by the neuroscientific community.

The data archives and systems closest to *brainlife.io* are the INDI,[98,99] OpenNeuro.org,[50] DANDI,[100] BossDB,[101] DataLad,[49] NITRC,[102] PING,[103] Can-CAM,[66] the Brain/MINDS project,[104] and LORIS.[105] The web services most related to the current work are NeuroQuery,[106] NeuroScout,[107] CBRAIN,[108] NeuroDesk,[109] XNAT,[110] NEMAR,[111] EBRAINS [112], LONI, [113,114], the International Brain Lab data Instratructure [115], COINSTAC [116] and CONP [117]. Most projects are open-source and provide various degrees of data access. *brainlife.io* end-to-end integrated environment that brings researchers from raw data to Jupyter Notebooks and Tidy data tables while tracking data provenance automatically is unique. But many other projects exist and given the fast-growing landscape of neuroinformatics projects, we collected a table listing the major ones (see **Supplementary Table 6**). The International Neuroinformatics Coordinating Facility (INCF) also provides a list of major projects incf.org/infrastructure-portfolio. brainlife.io is one of the approved resources, as it complies with the INCF requirement for FAIR infrastructure.

Supplementary Results 6: Current limitations and future development to improve platform usability and data gravity

The following discussion will include descriptions of the resources available for getting started on brainlife.io, applications of *brainlife.io* to educational settings, the platform's strict data governance principles, increasing "data gravity" via *brainlife.io*, potential expansion of the platform, and the platform's current limitations.

One key limitation of the platform is the complexity of the workflows provided. As a result of the fast-paced growth of the project, brainlife.io makes available a large number of services and data processing workflows. The learning curve to fully utilize these workflows has increased over the past few years. An important future goal of the project will be to find mechanisms to simplify access to the primary workflows on brainlife.io (e.g., the most used data pipelines) via GUI. This is a primary goal for the upcoming years of the project. Another current challenge is the complexity of the App execution error interpretation. It is currently difficult for users to understand what went wrong during the execution of an App when the execution is not complete successfully. brainlife.io provides a unique feature by returning the standard output (both log and err) files from the execution of an App in a high-performance compute cluster. These standard output files can in principle be used to understand, why an App failed, yet, the GUI does not provide simple mechanisms to visualize nor interpret the error messages. We plan to develop mechanisms to both standard output file visualization and to interpret the type of messages clusters return so as to provide a simplified interpretation of the reason for a failure (e.g., a network interruption, a memory overflow problem, or similar technical events).

Improving the platform's automation and interoperability is part of the vision and sustainability plan. For example, despite the best efforts of App developers, errors occur (see **Supplementary Figure 3**). Currently, researchers only have simple interfaces that report technical output logs and error messages when Apps fail to process data, and parsing these messages requires expertise. Users are required to either contact the *brainlife.io* team or parse the error logs themselves. Planned improvements to *brainlife.io*'s error reporting interfaces will help users understand the sources of errors and find solutions. In addition to error identification, identifying the optimal set of processing steps or parameter sets at the beginning of a project can prove challenging. In addition, currently, researchers identify the optimal data processing steps by looking at existing documentation or videos. In the future, mechanisms that automatically identify processing steps can be implemented to suggest to researchers optimal ways to process their data (e.g. given what other researchers might have already implemented on the platform). Finally, planned improvements to expand the functionality of *brainlife.io* for more data modalities, including non-neuroimaging modalities. Specifically, active collaborations and grants have been approved in order to expand the infrastructure for MEG/EEG analysis, behavioral data analysis, and genomic analyses. Yet, ultimately the impact of the platform and project will depend also on the development of data analysis stream from the community for the community.

Supplementary Results 7: *brainlife.io* and the FAIR principles.

The FAIR data principles for data stewardship and management[118] are generally used as guidelines for any data-centric project. Recently, it has been proposed that a modern definition of neuroscience data should extend beyond measurements and data to include metadata and research, analysis and management software[9,119]. *brainlife.io* was built with the FAIR principles in mind and below, we pair each FAIR principle with the modern definition of neuroscience data. In the *brainlife.io* project, each principle is applied to multiple research assets, data derivatives, analysis software, and software services.

The three primary research assets pertaining to the *brainlife.io* project are (1) data, derivatives, and metadata, (2) processing applications and data analysis code, and (3) data and analysis management services are each made FAIR via the *brainlife.io* project.

Findable. Research data services available on brainlife.io such as data sets, processing App, web services and analysis code are either automatic or manual mechanisms to make them findable. brainlife.io assigns Digital-Objects-Identifiers (DOI) using DataCite as a partner project. DOIs are automatically assigned to publication records consisting of datasets, as well as versioned preprocessing and analysis software. These brainlife.io publication records are compliant with schema.org and as such are also compliant with Google Dataset search (https://datasetsearch.research.google.com). DOIs are also assigned to each published App.

Accessible. Data and metadata can be retrieved using a number of access methods via Web Interfaces and Command Line Interfaces. Metadata is also accessible programmatically via a web API. Metadata remains available even in the case that data must be removed (e.g., in cases of human subjects concerns). Authentication is necessary to access the data and users' identities are checked by humans to assure compliance with more restrictive data-access policies such as the GDPR. A full record of data management and processing is made accessible. So not just data or analysis streams are accessible but a full record reporting the provenance of each individual data product. The code underlying each processing App is accessible via GitHub, and can be modified or used via common GitHub mechanisms (push requests, pull requests). Previously published datasets can be downloaded to a local machine or copied to a new project.

Interoperable. Data can be submitted to *brainlife.io* either using standard file types such as NifTis, but also data from multiple vendors can be used to map the data to the BIDS standard and uploaded on the system using the brainlife.io/ezBIDS web tool. The brainlife.io/ezBIDS system allows data from multiple vendors and type of sequences to be mapped to the Brain Imaging Data Structure (BIDS) and from there to be pushed to *brainlife.io* Projects, to OpenNeuro.org or downloaded. Furthermore, datasets can be mapped from major archives and projects such as NKI, and OpenNeuro.org using DataLad.org. Finally, *brainlife.io* Apps on their own also use are FAIR, as they are publicly available both as services on brainlife.io and code implementing the services on GitHub. The Apps can be stored either on individual user or organization accounts or on the *brainlife.io* team GitHub account depending on the level of commitment of the app developer to maintaining the Apps. The *brainlife.io* team maintains a bl2bids (https://github.com/brainlife/abcd-spec/blob/master/hooks/bl2bids.py) and the BIDS Walked (https://github.com/brainlife/cli/blob/master/bids-walker.js) script that together allow mapping BIDS data types to *brainlife.io* DataTypes. As a result the BIDS standard is the data exchange approach used to increase data interoperability.

Reusable. The brainlife.io project has multiple aspects of its technology that is developed with a mindset focus of reuse. First, the whole platform is developed as open source and published on GitHub.com. Second, the data processing Applications are developed using a lightweight specification that is compatible with BIDS and can be easily used without brainlife.io interfaces on local computers or clusters. Finally, data assets can be shared within the platform across users and projects but also outside of the platform by downloading the data as BIDS-compliant datasets. Data derivatives, processing apps, and analysis notebooks can be accessed in multiple ways via web graphical user interfaces, command line interfaces, or directly via local download. Analysis notebooks in the form of Jupyter notebooks can be pushed to GitHub directly, allowing for instantaneous reuse by the broader community. Data pipelines can be copied and reused within a given project. All configuration parameters for each App are stored, allowing users to reuse previously defined optimal parameters for their given data. The *brainlife.io* publication modelis a key component to implementing a vision of an integrated project publication containing data, and preprocessing for future reuse.

Supplementary Table 1: Videos demonstrating use of platform.

Video Description	Youtube Link
Creating a project on brainlife.io	https://youtu.be/P2kz6E53nlo
Import dataset archived on OpenNeuro into a project from the Datasets tab	https://youtu.be/N3UXteQ3tu8
Find datasets on OpenNeuro and import to brainlife project	https://youtu.be/OZQyR9jLwYo
Uploading data into a brainlife project using the GUI	https://youtu.be/5RGo_jY4Oqc
Upload data via the command line interface (CLI)	https://youtu.be/PUTLXJJSBqQ
Upload data into a brainlife project using ezBIDS	https://youtu.be/KvhIHxzHsl4
Running Apps on brainlife	https://youtu.be/43yhZ1k6icQ
Define a pipeline rule for batch processing on brainlife	https://youtu.be/1CSdsf8czL8
Launch a Jupyter Notebook for analysis on brainlife	https://youtu.be/tJW6374BcpQ
Visualize provenance to create a data object on brainlife	https://youtu.be/NzUObf8_x7g
Reproduce the creation of a data object on brainlife locally	https://youtu.be/YMCFU0aQhvI
Publish a dataset on brainlife	https://youtu.be/aUvjuEihWJA

Supplementary Table 1. Table with list of youtube videos describing how to use the various features of the platform.

Supplementary Table 2: Jupyter notebooks for analyses performed.

Notebook Name	Topic	Analysis/Figure	Datatype(s)	Measure(s)	Github URL
blp-analysis-structural-mri-volume.ipynb	Structural morphometry	Validity, reliability, reproducibility, development, references	neuro/parc-stats	Cortical parcel volume, thickness, surface area, Fractional Anisotropy (FA), Axial Diffusivity (AD), Radial Diffusivity (RD), Mean Diffusivity (MD), Neurite density index (NDI), Orientation dispersion index (ODI), Isotropic volume fraction (IsoVF)	https://github.com/bacaron/bp-notebooks/bl_paper/blp-analysis-structural-mri-volume.ipynb
blp-analysis-diffusion-mri-tract-profiles.ipynb	Diffusion profilometry	Validity, reliability, reproducibility, development, references	neuro/tractmeasures	White matter tract Fractional Anisotropy (FA), Axial Diffusivity (AD), Radial Diffusivity (RD), Mean Diffusivity (MD), Neurite density index (NDI), Orientation dispersion index (ODI), Isotropic volume fraction (IsoVF)	https://github.com/bacaron/bp-notebooks/bl_paper/blp-analysis-diffusion-mri-tract-profiles.ipynb

blp-analysis-diffusion-mri-structural-connectivity.ipynb	Structural connectivity	Validity, reliability, reproducibility, development, references	neuro/network	Max node degree	https://github.com/bacaron/bp-notebooks/bl_paper/blp-analysis-diffusion-mri-structural-connectivity.ipynb
blp-analysis-functional-mri-functional-connectivity.ipynb	Functional connectivity	Validity, reliability, reproducibility, development, references	neuro/network	Within-network connectivity	https://github.com/bacaron/bp-notebooks/bl_paper/blp-analysis-functional-mri-functional-connectivity.ipynb
blp-analysis-functional-mri-gradienty.ipynb	Functional gradients	Validity, reliability, reproducibility, development, references	neuro/gradients	Distance of primary gradient	https://github.com/bacaron/bp-notebooks/bl_paper/blp-analysis-functional-mri-gradientsy.ipynb
blp-analysis-meeg-power-spectrum-density.ipynb	MEEG	Validity, reliability, reproducibility, development, references	neuro/meeg/psd	Peak alpha frequency, power spectrum density	https://github.com/bacaron/bp-notebooks/bl_paper/blp-analysis-meeg-power-spectrum-density.ipynb
blp-analysis-concussion-structural-mri.ipynb	Cortical diffusion	Clinical populations	neuro/parc-stats	Cortical parcel volume, thickness, surface area, Fractional Anisotropy (FA), Axial Diffusivity (AD), Radial Diffusivity (RD), Mean Diffusivity (MD), Neurite density index (NDI), Orientation dispersion index (ODI), Isotropic volume fraction (IsoVF)	https://github.com/bacaron/bp-notebooks/bl_paper/blp-analysis-concussion-structural-mri.ipynb
blp-analysis-inherited-retinal-disease.ipynb	Diffusion profilometry, optical coherence tomography (OCT)	Clinical populations	neuro/tractmeasures, neuro/microperimetry	White matter tract Fractional Anisotropy (FA), Photoreceptor thickness	https://github.com/bacaron/bp-notebooks/bl_paper/blp-analysis-inherited-retinal-disease.ipynb
blp-analysis-usage-statistics.ipynb	Platform usage statistics	NA	NA	NA	https://github.com/bacaron/bp-notebooks/bl_paper/blp-analysis-usage-statistics.ipynb

Supplementary Table 2. Description and web-links to the open-source code used for each analysis outlined previously in the form of individual Jupyter Notebooks.

Supplementary Table 3: Preprocessing Apps used for the experiments.

Name	Brainlife DOI	Github Repository
Anatomically Constrained Tractography using precomputed 5tt & CSD	10.25663/brainlife.app.297	bacaron/app-mrtrix3-act
mrtrix3 - WMC Anatomically Constrained Tractography (ACT)	10.25663/brainlife.app.319	brainlife/app-mrtrix3-act
Compile tract macro-structural and profile data	10.25663/brainlife.app.397	brainlife/app-compile-macro-micro-tract-stats
Compute summary statistics of diffusion measures from subcortical segmentation	10.25663/brainlife.app.389	brainlife/app-freesurfer-stats
Compute summary statistics of diffusion measures mapped to the cortical surface - Deprecated Surface	10.25663/brainlife.app.383	brainlife/app-cortex-tissue-mapping-stats
Conmat 2 Network	10.25663/brainlife.app.393	filipinascimento/bl-conmat2network
Convert network neuro matrix to conmat	10.25663/brainlife.app.335	brainlife/app-network-matrices-2-mat
Cortex Tissue Mapping (Native & Template Space)	10.25663/brainlife.app.379	brainlife/app-cortex-tissue-mapping
Fit Constrained Deconvolution Model for Tracking	10.25663/brainlife.app.238	bacaron/app-mrtrix3-act
Freesurfer	10.25663/bl.app.0	brainlife/app-freesurfer
Freesurfer Statistics	10.25663/brainlife.app.272	brainlife/app-freesurfer-stats
FSL Anat (T1)	10.25663/brainlife.app.273	brainlife/app-fsl-anat
Align T1 to ACPC Plane (HCP-based)	10.25663/bl.app.99	brainlife/app-hcp-acpc-alignment
FSL Anat (T2)	10.25663/brainlife.app.350	brainlife/app-fsl-anat
FSL Brain Extraction (BET) on DWI	10.25663/brainlife.app.163	brainlife/app-FSLBET
mrtrix3 preprocess	10.25663/bl.app.68	brainlife/validator-neuro-dwi
Multi-Atlas Transfer Tool (w/surface output)	10.25663/bl.app.23	faskowit/app-multiAtlasTT
Noddi Amico	10.25663/brainlife.app.365	brainlife/app-noddi-amico
Parcellation Statistics - Surface - Deprecated Datatype	10.25663/brainlife.app.464	brainlife/app-freesurfer-stats
Remove Tract Outliers	10.25663/brainlife.app.195	brainlife/validator-neuro-wmc
Tissue-type segmentation	10.25663/brainlife.app.239	brainlife/app-mrtrix3-5tt
Tract Analysis Profiles	10.25663/brainlife.app.361	brainlife/app-tractanalysisprofiles
Tractography quality check	10.25663/brainlife.app.189	brainlife/app-tractographyQualityCheck
White Matter Anatomy Segmentation	10.25663/brainlife.app.188	brainlife/validator-neuro-wmc
Align T2 to ACPC Plane (HCP-based)	10.25663/brainlife.app.116	brainlife/app-hcp-acpc-alignment/tree/1.4
fMRIPrep - Volume Output	10.25663/brainlife.app.160	brainlife/app-fmriprep/tree/20.2.3-2
pRFs / Benson14-Retinotopy - Deprecated	10.25663/brainlife.app.187	davhunt/app-benson14-retinotopy/tree/master
Segment thalamic nuclei	10.25663/brainlife.app.222	brainlife/app-segment-thalamic-nuclei/tree/v1.0
Track The Human Optic RAdiation (THORA): Contrack - Eccentricity	10.25663/brainlife.app.252	brainlife/app-contrack-optic-radiation/tree/v1.1
Automated Segmentation of	10.25663/brainlife.app.262	svincibo/app-ashs-segment/tree/master

Hippocampal Subfields (ASHS)		
fMRIPrep - Surface Output	10.25663/brainlife.app.267	brainlife/app-fmriprep/tree/20.2.1
FSL DTIFIT	10.25663/brainlife.app.292	brainlife/app-fslDTIFIT/tree/v1.1
fMRI Timeseries Extraction	10.25663/brainlife.app.369	faskowit/app-fmri-2-mat/tree/0.1.6
Structural Connectome MRTrix3 (SCMRT) - No labels or weights	10.25663/brainlife.app.395	brainlife/app-sift2-connectome-generation/tree/nosift2_v1.2_centers_netneuro
Generate Visual Regions of Interest Binned by Eccentricity Estimates (Benson Atlas) - Diffusion Space	10.25663/brainlife.app.414	brainlife/app-roiGenerator/tree/visual-white-matter-eccentricity-dwi-v1.2
dsi-studio-atk	10.25663/brainlife.app.423	frankyeh/dsi-studio-atk/tree/master
Apply Maxwell filter on MEG signals using MNE-python	10.25663/brainlife.app.476	brainlife/app-maxwell-filter/tree/master
Compute summary statistics of diffusion measures mapped to cortical surface	10.25663/brainlife.app.483	brainlife/app-cortex-tissue-mapping-stats/tree/updated-surface-dtype-v1.1
Split MEG file	10.25663/brainlife.app.529	guiomar/app-meg-split-fif/tree/main
PSD: Power Spectral Density (Welch method)	10.25663/brainlife.app.530	guiomar/app-psd/tree/main
Find frequency peak of PSD data	10.25663/brainlife.app.531	guiomar/app-peak-frequency/tree/master
Time series to network	10.25663/brainlife.app.532	filipinascimento/bl-timeseries2network/tree/0.2
Connectivity Gradients	10.25663/brainlife.app.574	anibalsolon/app-connectivity-gradient/tree/main
Average channels	10.25663/brainlife.app.599	guiomar/app-average-channels/tree/main

Supplementary Table 3. Description and web links to the open-source code and open cloud services used to perform the evaluation experiments described in the main article.

Supplementary Table 4. Validity and reliability correlation tables.

Modality	Measure	Analysis	Parcellation	r	rmse
Structural MRI	Cortical thickness	Validity	Destrieux	0.8667	0.2332
"	Cortical surface area	Validity	Destrieux	0.9774	173.9724
"	Cortical volume	Validity	Destrieux	0.9817	570.543
"	Cortical thickness	Reliability	Destrieux	0.9569	0.121
"	Cortical surface area	Reliability	Destrieux	0.9930	97.4636
"	Cortical volume	Reliability	Destrieux	0.9948	2378.1114
"	Cortical thickness	Validity	hcp-mmp	0.8449	0.2416
"	Cortical surface area	Validity	hcp-mmp	0.9835	78.1686
"	Cortical volume	Validity	hcp-mmp	0.9727	265.6
"	Cortical thickness	Reliability	hcp-mmp	0.9402	0.1394
"	Cortical surface area	Reliability	hcp-mmp	0.9952	41.7407
"	Cortical volume	Reliability	hcp-mmp	0.9933	123.118
Diffusion MRI	Tract AD	Validity	wma	0.9572	0.0309
"	Tract FA	Validity	wma	0.9515	0.0181
"	Tract MD	Validity	wma	0.9167	0.0200
"	Tract RD	Validity	wma	0.9817	0.0228
"	Tract AD	Reliability	wma	0.9204	0.0402
"	Tract FA	Reliability	wma	0.9312	0.0167
"	Tract MD	Reliability	wma	0.806	0.0292
"	Tract RD	Reliability	wma	0.8447	0.0282
Functional MRI	Node connectivity	Validity	Yeo17	0.8853	0.1219
"	Node connectivity	Reliability	Yeo17	0.7264	0.1889
"	Primary gradient	Validity	Shaffer400	0.5934	0.0358
"	Primary gradient	Reliability	Shaffer400	0.8496	0.0259
MEEG	Peak alpha frequency	Validity	NA	0.9385	0.2964
"	Peak alpha frequency	Reliability	NA	0.8484	0.4751

Supplementary Table 4. Pearson correlation (*r*) and root mean square error (*rmse*) for all validity and reliability experiments performed.

Supplementary Table 5: Resources for data storage, archiving, and computational analysis.

Location(s)	Archive Name	Web URL	Type	Archive Representative	Data Modality (-ies)	Type of access	Reference (publication)
U.S.A	BRAIN Initiative Cell Census Network (BICCN)	www.biccn.org/	service registry	Multiple; the Allen Institute has an NIH grant to build and host this site, through the Brain Cell Data Center (BCDC)	human, mouse; single cell RNA-Seq, Patch-Seq, cell morphologies, electrophysiological recordings (NWB files), multiple histological image modalities, mFISH		
US BRAIN	BICCN Single Cell Portal	singlecell.broadinstitute.org/single_cell	service registry	Broad Institute scp-support@broadinstitute.zendesk.com	Multiple single cell datasets	N/A	
US BRAIN	OpenNeuro.org	OpenNeuro.org	Archive	Russ Poldrack	human MRI, PET, EEG,		
US BRAIN	DABI archive	dabi.loni.usc.edu/home	Archive	TOGA, ARTHUR W	EEG, MEG, iEEG		
US BRAIN	Allen Brain Map	portal.brain-map.org	service registry	Allen Institute - multiple teams involved	human, mouse, rhesus macaque		
US BRAIN	DANDI	www.dandiarchive.org/	Archive	Satrajit Ghosh	Neurophysiology (EPhys, ICEphys, Ophys)		
US BRAIN	NeMO	nemoarchive.org/	Archive	Owen R. White	Multi-omics data		
US BRAIN	Brain Image Library (BIL)	www.brainimagelibrary.org/	service registry	ROPELEWSKI, ALEXANDER J	Brain imaging data		
US BRAIN	BossDB	bossdb.org/	Archive	WESTER, BROCK A.	EM		
US BRAIN	MiCRONS Explorer	microns-explorer.org/	web-service	Multiple	EM		
US BRAIN	[their main site]	www.braininitiative.org/resources/	service registry		aggregator		
US BRAIN	brainlife.io	brainlife.io	computational platforms	Franco Pestilli	MRI/EEG/MEG	Governed via license	
Australian Initiative		neurodesk.org	web-service				
Japan Initiative	SRPBS	www.cns.atr.jp/decnefpro/	service registry	Saori Tanaka, Mitsuo Kawato	Brain imaging data		
Japan Initiative	Brain/MINDS Beyond	mriportal.umin.jp/	service registry	Kiyoto Kasai, Takashi Hanakawa, Saori Tanaka	Brain imaging data		

Japan Initiative	Brain/MINDS	www.brainminds.riken.jp/	service registry	Alex Woodward	Marmoset atlas, fMRI, dMRI, tracer, gene expression	Open to collaborators	
China Initiative	Linked Brain Data	www.linked-brain-data.org/	service registry				
Korea Initiative	Korea Brain Initiative	kbrain-map.kbri.re.kr:8080/	service registry	Sung-Jin Jeong	mouse; single cell RNA-Seq, EM data (current); omics data, behavioural data, electrophysiology data (in future)		
European Human Brain Project	EBRAINS	ebrains.eu/	service registry	Jan Bjaalie	Brain imaging data, omics data, behavioural data, electrophysiology data, models etc	Closed	
Canadian Open Neuroscience Platform	CONP	conp.ca/	service registry	CONP committee	Brain imaging data, omics data, behavioural data, electrophysiology data, models etc	Governed via license	
BlueBrainProject		channelpedia.epfl.ch/	service registry				
DataLad		datasets.datalad.org/	service registry			Fully open (CC-00)	
NITRC			service registry				
USA	WebPlotDigitizer	automeris.io/WebPlotDigitizer/	web-service	Ankit Rohatgi			
USA	Brain Map Database	brainmap.org	web-service	Peter Fox	Brain Imaging data	Governed via license	
USA	NeuroSynth Database	neurosnyth.org	web-service	Alejandro de la Vega	Brain Imaging data	Fully open (CC-00)	
France	NeuroQuery	https://neuroquery.org	web-service	INRIA/ Jérôme Dockès	Brain Imaging data	Fully open (CC-00)	
	OSF	osf.io	Archive		Unspecified / Open	Unspecified	
U.S.A.	COINSTAC	https://coinstac.org/	Downloadable	Georgia State University	Brain Imaging Data	Unspecified	

Supplementary Table 5. Description and web links to the many available platforms and services for increasing data gravity in the neuroimaging field.

Supplementary Table 6: Processed dataset published as part of this article.

Project	DOI	Brainlife Publication URL
Human Connectome Young Adult - Test - Retest	https://doi.org/10.25663/brainlife.pub.38	https://brainlife.io/pub/640a3da8c538c16a826f912e
Human Connectome Young Adult - Full Dataset	https://doi.org/10.25663/brainlife.pub.40	https://brainlife.io/pub/640a3f9dc538c16a826f9b1a
Cambridge Centre for Ageing and Neuroscience - Full Dataset	https://doi.org/10.25663/brainlife.pub.39	https://brainlife.io/pub/640a3f0cc538c16a826f9648
MEG [fif] Cam-Can	https://doi.org/10.25663/brainlife.pub.41	https://brainlife.io/pub/640a40fec538c16a826fa468
MEG [fif] Run1 vs Run2	https://doi.org/10.25663/brainlife.pub.42	https://brainlife.io/pub/640a4155c538c16a826fa5b9
MEG [fif] CamCan-maxfilt	https://doi.org/10.25663/brainlife.pub.43	https://brainlife.io/pub/640a41abc538c16a826fa6e6
ASHS Segmentation of Hippocampal Subfields - Replication derivatives	https://doi.org/10.25663/brainlife.pub.44	https://brainlife.io/pub/640a4267c538c16a826fb09a

Supplementary Table 6. Table with list of all platform services, name, scope, service URL (pointer to brainlife page if available as direct URL) and github URL for code.

References

1. Calhoun, V. D., Liu, J. & Adali, T. A review of group ICA for fMRI data and ICA for joint inference of imaging, genetic, and ERP data. *Neuroimage* **45**, S163–72 (2009).
2. Poldrack, R. A., Gorgolewski, K. J. & Varoquaux, G. Computational and Informatic Advances for Reproducible Data Analysis in Neuroimaging. *Annu. Rev. Biomed. Data Sci.* (2019) doi:10.1146/annurev-biodatasci-072018-021237.
3. Hériché, J.-K., Alexander, S. & Ellenberg, J. Integrating Imaging and Omics: Computational Methods and Challenges. *Annu. Rev. Biomed. Data Sci.* **2**, 175–197 (2019).
4. Shen, L. & Thompson, P. M. Brain Imaging Genomics: Integrated Analysis and Machine Learning. *Proc. IEEE Inst. Electr. Electron. Eng.* **108**, 125–162 (2020).
5. McPherson, B. C. & Pestilli, F. A single mode of population covariation associates brain networks structure and behavior and predicts individual subjects' age. *Commun Biol* **4**, 943 (2021).
6. Deslauriers-Gauthier, S. *et al.* White matter information flow mapping from diffusion MRI and EEG. *Neuroimage* **201**, 116017 (2019).
7. Wirsich, J., Amico, E., Giraud, A.-L., Goñi, J. & Sadaghiani, S. Multi-timescale hybrid components of the functional brain connectome: A bimodal EEG-fMRI decomposition. *Netw Neurosci* **4**, 658–677 (2020).
8. Engemann, D. A. *et al.* Combining magnetoencephalography with magnetic resonance imaging enhances learning of surrogate-biomarkers. *Elife* **9**, (2020).
9. Eke, D. O. *et al.* International data governance for neuroscience. *Neuron* (2021) doi:10.1016/j.neuron.2021.11.017.
10. Stewart, C. A. *et al.* Jetstream: A self-provisioned, scalable science and engineering cloud environment. (2015) doi:10.1145/2792745.2792774.
11. Hancock, D. Y. *et al.* Jetstream2: Accelerating cloud computing via Jetstream. in *Practice and Experience in Advanced Research Computing* 1–8 (Association for Computing Machinery, 2021).
12. Dale, A., Fischl, B. & Sereno, M. I. Cortical Surface-Based Analysis: I. Segmentation and Surface Reconstruction. *Neuroimage* **9**, 179–194 (1999).
13. Fischl, B., Sereno, M. I. & Dale, A. Cortical Surface-Based Analysis: II: Inflation, Flattening, and a Surface-Based Coordinate System. *Neuroimage* **9**, 195–207 (1999).
14. Desikan, R. S. *et al.* An automated labeling system for subdividing the human cerebral cortex on MRI scans into gyral based regions of interest. *Neuroimage* **31**, 968–980 (2006).
15. Fischl, B., Liu, A. & Dale, A. M. Automated manifold surgery: constructing geometrically accurate and topologically correct models of the human cerebral cortex. *IEEE Medical Imaging* **20**, 70–80 (2001).
16. Fischl, B. *et al.* Whole brain segmentation: automated labeling of neuroanatomical structures in the human brain. *Neuron* **33**, 341–355 (2002).
17. Fischl, B. *et al.* Sequence-independent segmentation of magnetic resonance images. *Neuroimage* **23**, S69–S84 (2004).
18. Fischl, B., Sereno, M. I., Tootell, R. B. H. & Dale, A. M. High-resolution intersubject averaging and a coordinate system for the cortical surface. *Hum. Brain Mapp.* **8**, 272–284 (1999).
19. Fischl, B. *et al.* Automatically Parcellating the Human Cerebral Cortex. *Cereb. Cortex* **14**, 11–22 (2004).
20. Han, X. *et al.* Reliability of MRI-derived measurements of human cerebral cortical thickness: The effects of field strength, scanner upgrade and manufacturer. *Neuroimage* **32**, 180–194 (2006).
21. Jovicich, J. *et al.* Reliability in multi-site structural MRI studies: Effects of gradient non-linearity correction on phantom and human data. *Neuroimage* **30**, 436–443 (2006).
22. Kuperberg, G. R. *et al.* Regionally localized thinning of the cerebral cortex in Schizophrenia. *Arch. Gen. Psychiatry* **60**, 878–888 (2003).
23. Reuter, M., Schmansky, N. J., Rosas, H. D. & Fischl, B. Within-Subject Template Estimation for Unbiased Longitudinal Image Analysis. *Neuroimage* **61**, 1402–1418 (2012).
24. Reuter, M. & Fischl, B. Avoiding Asymmetry-Induced Bias in Longitudinal Image Processing. *Neuroimage* **57**, 19–21 (2011).
25. Reuter, M., Rosas, H. D. & Fischl, B. Highly Accurate Inverse Consistent Registration: A Robust Approach. *Neuroimage* **53**, 1181–1196 (2010).
26. Salat, D. *et al.* Thinning of the cerebral cortex in aging. *Cereb. Cortex* **14**, 721–730 (2004).
27. Segonne, F. *et al.* A hybrid approach to the skull stripping problem in MRI. *Neuroimage* **22**, 1060–1075 (2004).
28. Segonne, F., Pacheco, J. & Fischl, B. Geometrically accurate topology-correction of cortical surfaces using nonseparating loops. *IEEE Trans. Med. Imaging* **26**, 518–529 (2007).
29. Tournier, J.-D., Calamante, F. & Connelly, A. MRtrix: Diffusion tractography in crossing fiber regions. *Int. J. Imaging Syst. Technol.* **22**, 53–66 (2012).
30. Tournier, J.-D. *et al.* MRtrix3: A fast, flexible and open software framework for medical image processing and visualisation. *Neuroimage* **202**, 116137 (2019).

31. Esteban, O. *et al.* fMRIPrep: a robust preprocessing pipeline for functional MRI. *Nat. Methods* **16**, 111–116 (2019).
32. Cieslak, M. *et al.* QSIPrep: an integrative platform for preprocessing and reconstructing diffusion MRI data. *Nat. Methods* **18**, 775–778 (2021).
33. Developers, S. *SingularityCE 3.8.3*. (2021). doi:10.5281/zenodo.5564915.
34. Merkel, D. Docker: lightweight Linux containers for consistent development and deployment. *Linux J.* **2014**, 2 (2014).
35. DataCite Schema. *DataCite Schema* https://schema.datacite.org/meta/kernel-4.1/index.html.
36. Gonzalez-Beltran, A. N. *et al.* Data discovery with DATS: exemplar adoptions and lessons learned. *J. Am. Med. Inform. Assoc.* **25**, 13–16 (2018).
37. Gonzalez-Beltran, A. & Rocca-Serra, P. *biocaddie/WG3-MetadataSpecifications: DataMed DATS specification v2.2 - NIH BD2K bioCADDIE*. (2017). doi:10.5281/zenodo.438337.
38. Caron, B. *et al.* Collegiate athlete brain data for white matter mapping and network neuroscience. *Sci Data* **8**, 56 (2021).
39. Bertò, G. *et al.* Classifyber, a robust streamline-based linear classifier for white matter bundle segmentation. *Neuroimage* **224**, 117402 (2021).
40. Sharmin, N., Olivetti, E. & Avesani, P. White Matter Tract Segmentation as Multiple Linear Assignment Problems. *Front. Neurosci.* **11**, 754 (2017).
41. Vinci-Booher, S., Caron, B., Bullock, D., James, K. & Pestilli, F. Development of white matter tracts between and within the dorsal and ventral streams. *Brain Struct. Funct.* **227**, 1457–1477 (2022).
42. Kurzawski, J. W., Mikellidou, K., Morrone, M. C. & Pestilli, F. The visual white matter connecting human area prostriata and the thalamus is retinotopically organized. *Brain Struct. Funct.* **225**, 1839–1853 (2020).
43. Sani, I. *et al.* The human endogenous attentional control network includes a ventro-temporal cortical node. *Nat. Commun.* **12**, 360 (2021).
44. Allen, E. J. *et al.* A massive 7T fMRI dataset to bridge cognitive neuroscience and artificial intelligence. *Nat. Neurosci.* **25**, 116–126 (2022).
45. Puzniak, R. J. *et al.* CHIASM, the human brain albinism and achiasma MRI dataset. *Sci Data* **8**, 308 (2021).
46. Hanekamp, S. *et al.* White matter alterations in glaucoma and monocular blindness differ outside the visual system. *Sci. Rep.* **11**, 6866 (2021).
47. Cheng, H. *et al.* Denoising diffusion weighted imaging data using convolutional neural networks. *PLoS One* **17**, e0274396 (2022).
48. Gorgolewski, K. J. *et al.* The brain imaging data structure, a format for organizing and describing outputs of neuroimaging experiments. *Sci Data* **3**, 160044 (2016).
49. Halchenko, Y. *et al.* DataLad: distributed system for joint management of code, data, and their relationship. *J. Open Source Softw.* **6**, 3262 (2021).
50. Markiewicz, C. J. *et al.* The OpenNeuro resource for sharing of neuroscience data. *Elife* **10**, (2021).
51. Nooner, K. B. *et al.* The NKI-Rockland Sample: A Model for Accelerating the Pace of Discovery Science in Psychiatry. *Front. Neurosci.* **6**, 152 (2012).
52. Tobe, R. H. *et al.* A longitudinal resource for studying connectome development and its psychiatric associations during childhood. *Sci Data* **9**, 300 (2022).
53. Di Martino, A. *et al.* The autism brain imaging data exchange: towards a large-scale evaluation of the intrinsic brain architecture in autism. *Mol. Psychiatry* **19**, 659–667 (2014).
54. Dean, J. & Ghemawat, S. MapReduce: simplified data processing on large clusters. *Commun. ACM* **51**, 107–113 (2008).
55. Kluyver, T. *et al.* Jupyter Notebooks – a publishing format for reproducible computational workflows. in *Positioning and Power in Academic Publishing: Players, Agents and Agendas* (eds. Loizides, F. & Scmidt, B.) 87–90 (IOS Press, 2016).
56. Perez, F. & Granger, B. E. IPython: A System for Interactive Scientific Computing. *Comput. Sci. Eng.* **9**, 21–29 (2007).
57. Wickham, H. Tidy Data. *J. Stat. Softw.* **59**, 1–23 (2014).
58. Avesani, P. *et al.* The open diffusion data derivatives, brain data upcycling via integrated publishing of derivatives and reproducible open cloud services. *Sci Data* **6**, 69 (2019).
59. Glatard, T. *et al.* Boutiques: a flexible framework to integrate command-line applications in computing platforms. *Gigascience* **7**, (2018).
60. Glatard, T. *et al.* A virtual imaging platform for multi-modality medical image simulation. *IEEE Trans. Med. Imaging* **32**, 110–118 (2013).
61. Deelman, E. *et al.* Pegasus in the Cloud: Science Automation through Workflow Technologies. *IEEE Internet Comput.* **20**, 70–76 (2016).
62. Alexander, L. M. *et al.* An open resource for transdiagnostic research in pediatric mental health and learning disorders. *Sci Data* **4**, 170181 (2017).
63. National Academies of Sciences, Engineering *et al. Understanding Reproducibility and Replicability*. (National Academies Press (US), 2019).
64. Kelley, T. L. Interpretation of educational measurements. **353**, (1927).

65. Van Essen, D. C. *et al.* The WU-Minn Human Connectome Project: an overview. *Neuroimage* **80**, 62–79 (2013).
66. Shafto, M. A. *et al.* The Cambridge Centre for Ageing and Neuroscience (Cam-CAN) study protocol: a cross-sectional, lifespan, multidisciplinary examination of healthy cognitive ageing. *BMC Neurol.* **14**, 204 (2014).
67. Casey, B. J. *et al.* The Adolescent Brain Cognitive Development (ABCD) study: Imaging acquisition across 21 sites. *Dev. Cogn. Neurosci.* **32**, 43–54 (2018).
68. Yeo, B. T. T. *et al.* The organization of the human cerebral cortex estimated by intrinsic functional connectivity. *J. Neurophysiol.* **106**, 1125–1165 (2011).
69. Bethlehem, R. A. I. *et al.* Dispersion of functional gradients across the adult lifespan. *Neuroimage* **222**, 117299 (2020).
70. Margulies, D. S. *et al.* Situating the default-mode network along a principal gradient of macroscale cortical organization. *Proc. Natl. Acad. Sci. U. S. A.* **113**, 12574–12579 (2016).
71. Taulu, S. & Kajola, M. Presentation of electromagnetic multichannel data: The signal space separation method. *J. Appl. Phys.* **97**, 124905 (2005).
72. Taulu, S. & Simola, J. Spatiotemporal signal space separation method for rejecting nearby interference in MEG measurements. *Phys. Med. Biol.* **51**, 1759–1768 (2006).
73. Botvinik-Nezer, R. *et al.* Variability in the analysis of a single neuroimaging dataset by many teams. *Nature* **582**, 84–88 (2020).
74. Noble, S., Scheinost, D. & Constable, R. T. A decade of test-retest reliability of functional connectivity: A systematic review and meta-analysis. *Neuroimage* **203**, 116157 (2019).
75. Esteban, O. *et al.* MRIQC: Advancing the automatic prediction of image quality in MRI from unseen sites. *PLoS One* **12**, e0184661 (2017).
76. Yeh, F.-C. Shape analysis of the human association pathways. *Neuroimage* **223**, 117329 (2020).
77. Cameron, C. *et al.* Towards automated analysis of connectomes: The configurable pipeline for the analysis of connectomes (C-PAC). *Front. Neuroinform.* **7**, (2013).
78. Fischl, B. FreeSurfer. *Neuroimage* **62**, 774–781 (2012).
79. Keshavan, A., Yeatman, J. D. & Rokem, A. Combining Citizen Science and Deep Learning to Amplify Expertise in Neuroimaging. *Front. Neuroinform.* **13**, 29 (2019).
80. Yanni, S. E. *et al.* Normative reference ranges for the retinal nerve fiber layer, macula, and retinal layer thicknesses in children. *Am. J. Ophthalmol.* **155**, 354–360.e1 (2013).
81. Esteban, O. *et al.* Crowdsourced MRI quality metrics and expert quality annotations for training of humans and machines. *Sci Data* **6**, 30 (2019).
82. Pedregosa, F. *et al.* Scikit-learn: Machine Learning in Python. *J. Mach. Learn. Res.* **12**, 2825–2830 (2011).
83. Fukutomi, H. *et al.* Neurite imaging reveals microstructural variations in human cerebral cortical gray matter. *Neuroimage* **182**, 488–499 (2018).
84. Ho, T. C. *et al.* Effects of sensitivity to life stress on uncinate fasciculus segments in early adolescence. *Soc. Cogn. Affect. Neurosci.* **12**, 1460–1469 (2017).
85. Hanson, J. L., Knodt, A. R., Brigidi, B. D. & Hariri, A. R. Lower structural integrity of the uncinate fasciculus is associated with a history of child maltreatment and future psychological vulnerability to stress. *Dev. Psychopathol.* **27**, 1611–1619 (2015).
86. Yushkevich, P. A. *et al.* Automated volumetry and regional thickness analysis of hippocampal subfields and medial temporal cortical structures in mild cognitive impairment. *Hum. Brain Mapp.* **36**, 258–287 (2015).
87. Karcher, N. R. & Barch, D. M. The ABCD study: understanding the development of risk for mental and physical health outcomes. *Neuropsychopharmacology* **46**, 131–142 (2021).
88. Yoshimine, S. *et al.* Age-related macular degeneration affects the optic radiation white matter projecting to locations of retinal damage. *Brain Struct. Funct.* **223**, 3889–3900 (2018).
89. Ogawa, S. *et al.* White matter consequences of retinal receptor and ganglion cell damage. *Invest. Ophthalmol. Vis. Sci.* **55**, 6976–6986 (2014).
90. Malania, M., Konrad, J., Jägle, H., Werner, J. S. & Greenlee, M. W. Compromised Integrity of Central Visual Pathways in Patients With Macular Degeneration. *Invest. Ophthalmol. Vis. Sci.* **58**, 2939–2947 (2017).
91. Sherbondy, A. J., Dougherty, R. F., Ben-Shachar, M., Napel, S. & Wandell, B. A. ConTrack: finding the most likely pathways between brain regions using diffusion tractography. *J. Vis.* **8**, 15.1–16 (2008).
92. Yeatman, J. D., Dougherty, R. F., Myall, N. J., Wandell, B. A. & Feldman, H. M. Tract profiles of white matter properties: automating fiber-tract quantification. *PLoS One* **7**, e49790 (2012).
93. Aydogan, D. B. & Shi, Y. Parallel Transport Tractography. *IEEE Trans. Med. Imaging* **40**, 635–647 (2021).
94. Baran, D. & Shi, Y. A novel fiber-tracking algorithm using parallel transport frames. in *ISMRM* (unknown, 2019).
95. Reer, A., Wiebe, A., Wang, X. & Rieger, J. W. FAIR human neuroscientific data sharing to advance AI driven research and applications: Legal frameworks and missing metadata standards. *Front. Genet.* **14**, 1086802 (2023).
96. Kozlov, M. NIH issues a seismic mandate: share data publicly. *Nature Publishing Group UK*

http://dx.doi.org/10.1038/d41586-022-00402-1 (2022) doi:10.1038/d41586-022-00402-1.
97. Infrastructure cards. https://www.incf.org/infrastructure-portfolio.
98. Biswal, B. B. *et al.* Toward discovery science of human brain function. *Proc. Natl. Acad. Sci. U. S. A.* **107**, 4734–4739 (2010).
99. Milham, M. P. Open neuroscience solutions for the connectome-wide association era. *Neuron* **73**, 214–218 (2012).
100. Halchenko, Y. *et al. dandi/dandi-cli: 0.46.2*. (2022). doi:10.5281/zenodo.7041535.
101. Hider, R., Jr *et al.* The Brain Observatory Storage Service and Database (BossDB): A Cloud-Native Approach for Petascale Neuroscience Discovery. *Front. Neuroinform.* **16**, 828787 (2022).
102. Kennedy, D. N., Haselgrove, C., Riehl, J., Preuss, N. & Buccigrossi, R. The NITRC image repository. *Neuroimage* **124**, 1069–1073 (2016).
103. Jernigan, T. L. *et al.* The Pediatric Imaging, Neurocognition, and Genetics (PING) Data Repository. *Neuroimage* **124**, 1149–1154 (2016).
104. Koike, S. *et al.* Brain/MINDS beyond human brain MRI project: A protocol for multi-level harmonization across brain disorders throughout the lifespan. *Neuroimage Clin* **30**, 102600 (2021).
105. Das, S., Zijdenbos, A. P., Harlap, J., Vins, D. & Evans, A. C. LORIS: a web-based data management system for multi-center studies. *Front. Neuroinform.* **5**, 37 (2011).
106. Dockès, J. *et al.* NeuroQuery, comprehensive meta-analysis of human brain mapping. *Elife* **9**, (2020).
107. de la Vega, A. *et al.* Neuroscout, a unified platform for generalizable and reproducible fMRI research. *Elife* **11**, (2022).
108. Sherif, T. *et al.* CBRAIN: a web-based, distributed computing platform for collaborative neuroimaging research. *Front. Neuroinform.* **8**, 54 (2014).
109. Renton, A. I. *et al.* Neurodesk: An accessible, flexible, and portable data analysis environment for reproducible neuroimaging. *bioRxiv* 2022.12.23.521691 (2022) doi:10.1101/2022.12.23.521691.
110. Marcus, D. S., Olsen, T. R., Ramaratnam, M. & Buckner, R. L. The Extensible Neuroimaging Archive Toolkit: an informatics platform for managing, exploring, and sharing neuroimaging data. *Neuroinformatics* **5**, 11–34 (2007).
111. Delorme, A. *et al.* NEMAR: an open access data, tools and compute resource operating on neuroelectromagnetic data. *Database* **2022**, (2022).
112. Schirner, M. *et al.* Brain simulation as a cloud service: The Virtual Brain on EBRAINS. *Neuroimage* **251**, 118973 (2022).
113. Rex, D. E., Ma, J. Q. & Toga, A. W. The LONI Pipeline Processing Environment. *Neuroimage* **19**, 1033–1048 (2003).
114. Elam, J. S. *et al.* The Human Connectome Project: A Retrospective. *Neuroimage* 118543 (2021).
115. International Brain Laboratory *et al.* A modular architecture for organizing, processing and sharing neurophysiology data. *Nat. Methods* (2023) doi:10.1038/s41592-022-01742-6.
116. Plis, S. M. *et al.* COINSTAC: A Privacy Enabled Model and Prototype for Leveraging and Processing Decentralized Brain Imaging Data. *Front. Neurosci.* **10**, 365 (2016).
117. Harding, R. J., Bermudez, P., Beauvais, M., Bellec, P. & Evans, A. C. The Canadian Open Neuroscience Platform – An Open Science Framework for the Neuroscience Community. (2022) doi:10.31219/osf.io/eh349.
118. Wilkinson, M. D. *et al.* The FAIR Guiding Principles for scientific data management and stewardship. *Sci Data* **3**, 160018 (2016).
119. Barker, M. *et al.* Introducing the FAIR Principles for research software. *Sci Data* **9**, 622 (2022).
