# Supplementary material for: brainlife.io: a decentralized and open-source cloud platform to support neuroscience research
Source: Nat Methods. 2024 Apr 11;21(5):809–13. doi: 10.1038/s41592-024-02237-2 (PMC11093740; doi:10.1038/s41592-024-02237-2)
Supplement: Supplementary file 2 — Reporting Summary [file 41592_2024_2237_MOESM2_ESM.pdf]

nature portfolio

Corresponding author(s): Franco Pesitlli
Last updated by author(s): 12/12/2023

Reporting Summary

Nature Portfolio wishes to improve the reproducibility of the work that we publish. This form provides structure for consistency and transparency in reporting. For further information on Nature Portfolio policies, see our Editorial Policies and the Editorial Policy Checklist.

Statistics

For all statistical analyses, confirm that the following items are present in the figure legend, table legend, main text, or Methods section.

n/a	Confirmed	
☐	☒	The exact sample size (*n*) for each experimental group/condition, given as a discrete number and unit of measurement
☐	☒	A statement on whether measurements were taken from distinct samples or whether the same sample was measured repeatedly
☐	☒	The statistical test(s) used AND whether they are one- or two-sided<br>*Only common tests should be described solely by name; describe more complex techniques in the Methods section.*
☐	☒	A description of all covariates tested
☒	☐	A description of any assumptions or corrections, such as tests of normality and adjustment for multiple comparisons
☐	☒	A full description of the statistical parameters including central tendency (e.g. means) or other basic estimates (e.g. regression coefficient) AND variation (e.g. standard deviation) or associated estimates of uncertainty (e.g. confidence intervals)
☐	☒	For null hypothesis testing, the test statistic (e.g. *F*, *t*, *r*) with confidence intervals, effect sizes, degrees of freedom and *P* value noted<br>*Give P values as exact values whenever suitable.*
☐	☒	For Bayesian analysis, information on the choice of priors and Markov chain Monte Carlo settings
☐	☒	For hierarchical and complex designs, identification of the appropriate level for tests and full reporting of outcomes
☐	☒	Estimates of effect sizes (e.g. Cohen's *d*, Pearson's *r*), indicating how they were calculated

Our web collection on statistics for biologists contains articles on many of the points above.

Software and code

Policy information about availability of computer code

Data collection	This article utilizes open data repositories all described in the methods section.
Data analysis	No commercial software were used for data analysis. All data analyses were made using open source code, developed by the project or from other open source projects. Software packages used to process the data described in the manuscript include, but may not be limited to: Freesurfer, FSL, AFNI, ANTs, fMRIPrep, JSON, python, pandas, numpy, scipy, seaborn, QSIPrep, Dipy, MRTrix3, Trekker, dsistudio, Nilearn, mne-python, connectome workbench, MatLab, Nibabel, pyAFQ, and MRIQC. All notebooks used to analyze the data can be found at https://github.com/bp-notebooks/bl_paper. All code supporting the platform can be found at https:/github.com/brainlife. Tables provided in Supplementary Information contain information about exact links to notebooks and platform code.

For manuscripts utilizing custom algorithms or software that are central to the research but not yet described in published literature, software must be made available to editors and reviewers. We strongly encourage code deposition in a community repository (e.g. GitHub). See the Nature Portfolio guidelines for submitting code & software for further information.

Data

Policy information about availability of data

All manuscripts must include a data availability statement. This statement should provide the following information, where applicable:

- Accession codes, unique identifiers, or web links for publicly available datasets
- A description of any restrictions on data availability
- For clinical datasets or third party data, please ensure that the statement adheres to our policy

All data derived and described in this paper are made available via the brainlife.io platform as "Publications". User data agreements are required for some projects, like data from the HCP, Cam-CAN, PING, ABCD, and HBN datasets. The Indiana University Acute Concussion Dataset and the Oxford University Choroideremia & Stargardt's Disease Dataset are parts of ongoing research projects and are not being released at this current time. All other datasets are made freely available via the brainlife.io platform. See Supplementary Table 6 for the brainlife.io/pubs [we have added one example data record (https://doi.org/10.25663/brainlife.pub.40) for the review process <the DOIs for the remaining data records will be added at publication>].

Human research participants

Policy information about studies involving human research participants and Sex and Gender in Research.

Reporting on sex and gender	We used open repositories and other secondary data that have their own policies for reporting. We did not analyze subjects by either sex or gender.
Population characteristics	For some analyses, we analyzed the relationship between brain properties and age. For others, we analyzed the relationship between brain properties and clinical disorder diagnosis. These specific analyses can be found in Figure 1 (a, d) of the manuscript. For no other analyses did we include covariates.
Recruitment	We did not recruit subjects as we used secondary data.
Ethics oversight	All ethics review was performed at the sites of the original data collectors. We performed only secondary data analysis and were not involved in recruiting subjects.

Note that full information on the approval of the study protocol must also be provided in the manuscript.

Field-specific reporting

Please select the one below that is the best fit for your research. If you are not sure, read the appropriate sections before making your selection.

☐ Life sciences ☒ Behavioural & social sciences ☐ Ecological, evolutionary & environmental sciences

For a reference copy of the document with all sections, see nature.com/documents/nr-reporting-summary-flat.pdf

Behavioural & social sciences study design

All studies must disclose on these points even when the disclosure is negative.

Study description	A multi-factoral study design was implemented using secondary data analyses. Some analyses involved using brain properties to test platform tool validity, reliability, and replicability. Other analyses involved using brain properties to identify those within specific clinical populations. All analyses were quantitative in scope.
Research sample	We used a large number of participants (~3000) in totum for all analyses from a variety of backgrounds (populations, scanner types). We used secondary data from many open projects, including the following: HCP, Cam-CAN, PING, ABCD, UPENN, and HBN. We have provided detailed descriptions of the data used from each cohort in the main manuscript online methods section.
Sampling strategy	We attempted to use all subjects available for each dataset. In certain situations, including the PING and ABCD datasets, only a subset of participants were used. For the PING, we only had access to the Siemen's scanner data at the time of analyses and manuscript preparation, leaving us with around 100 subjects. For the ABCD, we only used a random subset of ~1100 participants. Overall, because our results are not scientific in nature, these provided a large enough sample to demonstrate the platform's capabilities overall.
Data collection	We only performed secondary data analysis and did not collect new data for the study. Because of this, research blindness to experimental conditions or hypotheses were not required.
Timing	We only performed secondary data analysis and did not collect new data for the study.
Data exclusions	For the PING, we only had access to the Siemen's scanner data at the time of analyses and manuscript preparation, leaving us with around 100 subjects. For the ABCD, we only used a random subset of ~1100 participants. Overall, because our results are not

	scientific in nature, these provided a large enough sample to demonstrate the platform's capabilities overall. Otherwise no data were excluded.
Non-participation	We only performed secondary data analysis and did not collect new data for the study. No participants were involved.
Randomization	We only performed secondary data analysis and did not collect new data for the study.

Reporting for specific materials, systems and methods

We require information from authors about some types of materials, experimental systems and methods used in many studies. Here, indicate whether each material, system or method listed is relevant to your study. If you are not sure if a list item applies to your research, read the appropriate section before selecting a response.

Materials & experimental systems

n/a	Involved in the study	
☒	☐	Antibodies
☒	☐	Eukaryotic cell lines
☒	☐	Palaeontology and archaeology
☒	☐	Animals and other organisms
☒	☐	Clinical data
☒	☐	Dual use research of concern

Methods

n/a	Involved in the study	
☒	☐	ChIP-seq
☒	☐	Flow cytometry
☐	☒	MRI-based neuroimaging

Magnetic resonance imaging

Experimental design

Design type	We only performed secondary data analysis and did not collect new data for the study.
Design specifications	We only performed secondary data analysis and did not collect new data for the study.
Behavioral performance measures	We only performed secondary data analysis and did not collect new data for the study.

Acquisition

Imaging type(s)	functional, diffusion-weighted, anatomical, meg/eeg
Field strength	3 Tesla
Sequence & imaging parameters	Validity, reliability, reproducibility, replicability, developmental trends, & reference datasets<br>Human Connectome Project (HCP; Test-Retest, s1200-release) 23. Data from these projects were used to assess the validity, reliability, and reproducibility of the platform. They were used to assess the abilities of the platform to identify developmental trends in structural and functional measures, and they were used to generate reference datasets. Structural data (sMRI): The minimally-preprocessed structural Tlw and T2w images from the Human Connectome Project (HCP) from 1066 participants from the s1200 and 44 participants from the Test-Retest releases were used. Specifically, the 1.25 mm 'acpc_dc_restored' images generated from the Siemens 3T MRI scanner were used for all analyses involving the HCP. For most examinations, the already-processed Freesurfer output from HCP was used. Diffusion data (dMRI): To assess the validity of preprocessing on brainlife.io, the unprocessed dMRI data from 44 participants from the HCP Test dataset was used. For reliability and all remaining analyses, the minimally-preprocessed diffusion (dMRI) images from 1,066 participants from the s1200 and 44 participants from the Test-Retest releases from the 3T Siemens scanner were used. All processes incorporated the multi-shell acquisition data. Functional data (fMRI): For validation, the unprocessed resting-state functional MRI (fMRI) from 44 participants from the HCP Test dataset was compared to the minimally-preprocessed BOLD data provided by HCP. For reliability and all other analyses, the minimally-preprocessed BOLD data from 1,066 participants from the s1200 and 44 participants from the Test-Retest releases from the 3T Siemens scanner were used.<br>The Cambridge Centre for Ageing and Neuroscience (Cam-CAN) 7. The data from this project were used to assess the validity, reliability, and reproducibility of the platform and to assess the abilities of the platform to identify developmental trends of structural and functional measures, and to generate reference datasets. Structural data (sMRI): The unprocessed 1mm isotropic structural Tlw and T2w images from 652 participants from the Cambridge Centre for Ageing and Neuroscience (Cam-CAN) study were used. Diffusion data (dMRI): The unprocessed 2mm isotropic diffusion (dMRI) images from 652 participants from the Cambridge Centre for Ageing and Neuroscience (Cam-CAN) study were used. Functional data (fMRI): The 3mm x 3mm x 4mm unprocessed resting-state fMRI images from 652 participants from the Cambridge Centre for Ageing and Neuroscience (Cam-CAN) study were used. Electromagnetic data (MEG): The 1000 Hz resting-state filtered and unfiltered datasets from 652 participants from the Cambridge Centre for Ageing and Neuroscience (Cam-CAN) study were used.<br>Developmental trends & reference datasets<br>Pediatric Imaging, Neurocognition, and Genetics (PING) 11. The data from this project were used to assess the abilities of the platform to identify developmental trends of structural measures and to generate reference datasets. Structural

data (sMRI): The unprocessed 1.2 x 1.0 x 1.0 mm structural Tlw and the 1.0 mm isotropic T2w images from 110 participants from the Pediatric Imaging, Neurocognition, and Genetics (PING) study were used. Diffusion data (dMRI): The unprocessed 2mm isotropic diffusion (dMRI) images from 110 participants from the Pediatric Imaging, Neurocognition, and Genetics (PING) study were used.
Replicability datasets
Adolescent Brain Cognitive Development (ABCD) 8,28. Structural data (sMRI): The unprocessed 1mm isotropic structural Tlw and T2w images from a subset of 1,877 participants from the Adolescent Brain Cognitive Development (ABCD release-2.0.0) study were used. Diffusion data (dMRI): The unprocessed 1.77mm isotropic diffusion (dMRI) images from a subset of 1877 participants from the Adolescent Brain Cognitive Development (ABCD release-2.0.0) study were used. A single diffusion gradient shell was used for these experiments (b=3000s/msec2). Research approved by the University of Arkansas IRB (#2209425822).
Healthy Brain Network (HBN) 10. The data from this project were used to assess the abilities of the platform to replicate previously published findings via the assessment of the relationship between microstructural measures mapped to segmented uncinate fasciculi and self-reported early life stressors. Research approved by the University of Pittsburgh IRB (#PRO17060350). Structural data (sMRI): The 0.8 mm isotropic structural Tlw images from 42 participants from the Healthy Brain Network (HBN) study were used. Diffusion data (dMRI): The unprocessed 1.8 mm isotropic diffusion (dMRI) images from 42 participants from the CitiGroup Cornell Brain Imaging Center site of the Healthy Brain Network (H BN) study were used. Research approved by the University of Pittsburgh I RB (#PROl 7060350).
UPENN-PMC 29. The data from this project were used to assess the abilities of the platform to replicate previously published findings via the assessment of the performance of an automated hippocampal segmentation algorithm. All procedures were conducted under the approval of the Institutional Review Board at the University of Texas at Austin. Structural data (sMRI): The Tlw and T2w data were provided within the Automated Segmentation of Hippocampal Subfields (ASHS) atlas29.
Clinical-identification datasets
Indiana University Acute Concussion Dataset. The data from this project were used to assess the abilities of the platform to identify clinical populations via the mapping of microstructural measures to the cortical surface. Neuroimaging was performed at the Indiana University Imaging Research Facility, housed within the Department of Psychological and Brain Sciences with a 3-Tesla Siemens Prisma whole-body MRI using a 64-channel head coil. Within this study, 9 concussed athletes and 20 healthy athletes were included. Research approved by Indiana University (IRB: 906000405). Structural data (sMRI): High-resolution Tl-weighted structural volumes were acquired using an MPRAGE sequence: Tl = 900 ms, TE = 2.7 ms, TR = 1800 ms, flip angle = 9°, with 192 sagittal slices of 1.0 mm thickness, a field of view of 256 x 256 mm, and an isometric voxel size of 1.0 mm3. The total acquisition time was 4 minutes and 34 seconds. High-resolution T2-weighted structural volumes were also acquired: TE = 564 ms, TR = 3200 ms, flip angle = 120°, with 192 sagittal slices, a field of view of 240 x 256 mm, and an isometric voxel size of l.0mm3. Total acquisition time was 4 minutes 30 seconds. Diffusion data (dMRI): Diffusion data were collected using single-shot spin-echo simultaneous multi-slice (SMS) EPI (transverse orientation, TE = 92.00 ms, TR = 3,820 ms, flip angle = 78 degrees, isotropic 1.5 mm3 resolution; FOV = LR 228 mm x 228 mm x 144 mm; acquisition matrix MxP = 138 x 138. SMS acceleration factor = 4). This sequence was collected twice, one in the AP fold-over direction and the other in the PA fold-over direction, with the same diffusion gradient strengths and the number of diffusion directions: 30 diffusion directions at b = 1000 s/mm2, 60 diffusion directions at b = 1,750 s/mm2, 90 diffusion directions at b = 2,500 s/mm2, and 19 b = 0 s/mm2 volumes. The total acquisition time for both sets of dMRI sequences was 25 minutes and 58 seconds.
Oxford University Choroideremia & Stargardt's Disease Dataset. The data from this project was used to assess the abilities of the platform to identify clinical populations via mapping retinal-layer thickness via OCT and mapping of microstructural measures along optic radiation bundles segmented using visual field information (eccentricity). Neuroimaging was performed at the Wellcome Centre for Integrative Neuroimaging, Oxford with the Siemens 3T scanner. Research approved by the UK Health Regulatory Authority reference 17/LO/1540. Structural data (sMRI): High-resolution Tl-weighted anatomical volumes were acquired using an MPRAGE sequence: Tl = 904 ms, TE = 3.97 ms, TR = 1900 ms, flip angle = 8°, with 192 sagittal slices of 1.0 mm thickness, a field of view of 174 mm x 192 mm x 192 mm, and an isometric voxel size of 1.0 mm3. The total acquisition time was 5 minutes and 31 seconds. Diffusion data (dMRI): Diffusion data were collected using EPI (transverse orientation, TE = 92.00ms, TR = 3600 ms, flip angle = 78 degrees, 2.019 x 2.019 x 2.0 mm3 resolution; FOV = 210 mm x 220 mm x 158 mm; acquisition matrix MxP = 210 x 210, SMS acceleration factor = 3). This sequence was collected twice, one in the AP fold-over direction and the other in the PA fold-over direction. The PA fold-over scan contained 6 diffusion directions, 3 at b = 0 s/mm2 and 3 at b = 2000 s/mm2, and was used primarily for susceptibility-weighted corrections. The AP fold-over scan contained 105 diffusion directions, 5 at b = 0 mm/s2, 51 at b = 1000 mm/s2, and 49 at b = 2000 mm/s2. The total acquisition time for both sets of dMRI sequences was 7 minutes and 8 seconds.

Area of acquisition: Brain

Diffusion MRI: ☒ Used ☐ Not used

Parameters: A variety of diffusion MRI sources were used and are described above.

Preprocessing

Preprocessing software: We used a variety of processing software, including but not limited to: Freesurfer, FSL, AFNI, ANTs, fMRIPrep, JSON, python, pandas, numpy, scipy, seaborn, QSIPrep, Dipy, MRTrix3, Trekker, dsistudio, Nilearn, mne-python, connectome workbench, MatLab, Nibabel, pyAFQ, and MRIQC.

Normalization: Linear and non-linear regression were used.

Normalization template: Various templates were depending on the analysis. The FreeSurfer FS-average in MNI coordinates, the Yeo17 atlas and the Human Connectome Multimodal Parcellation

Noise and artifact removal: We performed motion correction, Eddy Currents Correction, bias field correction, max-filtering of MEG data

Volume censoring: n/a

Statistical modeling & inference

Model type and settings: n/a

Effect(s) tested: n/a

Specify type of analysis: ☐ Whole brain ☐ ROI-based ☒ Both

Anatomical location(s): multiple locations were used depending on the type of analysis performed to validate the brainlife.io platform

Statistic type for inference (See Eklund et al. 2016): n/a

Correction: FDR

Models & analysis

n/a	Involved in the study
☐	☒ Functional and/or effective connectivity
☐	☒ Graph analysis
☒	☐ Multivariate modeling or predictive analysis

Functional and/or effective connectivity: Pearson correlation

Graph analysis: Binarized data and Node Degree
